# Structure–activity relationships can be directly extracted from high-throughput crystallographic evaluation of fragment elaborations in crude reaction mixtures

**DOI:** 10.1039/d5sc04919a

**Published:** 2025-12-23

**Authors:** Harold Grosjean, Kate K. Fieseler, Rubén Sanchez-Garcia, Warren Thompson, Charlotte M. Deane, Frank von Delft, Philip C. Biggin

**Affiliations:** a Structural Bioinformatics and Computational Biochemistry, Department of Biochemistry, University of Oxford South Parks Road Oxford OX1 3QU UK; b Diamond Light Source Ltd Harwell Science and Innovation Campus, Didcot OX11 0QX UK; c Oxford Protein Informatics Group, Department of Statistics, University of Oxford Oxford OX1 3LB UK; d Research Complex at Harwell Harwell Science and Innovation Campus, Didcot OX11 0FA UK; e Centre for Medicines Discovery, University of Oxford Oxford OX3 7DQ UK; f Department of Biochemistry, University of Johannesburg Auckland Park 2006 South Africa

## Abstract

Fragment-based drug design offers multiple routes to advance from fragments. One approach is to build structure–activity relationships (SAR) from analogue series in direct-to-biology workflows. Analogues can be prepared by automated chemistry and tested as crude reaction mixtures (CRMs) without purification, but assay noise often leads to hit resynthesis, potentially discarding false negatives and reducing SAR dataset size. High-throughput (HT) X-ray crystallography has the potential to address these issues by resolving hits directly from 100s–1000s of CRMs. However, no systematic analytics exist for extracting SAR models from HT crystallographic evaluation of CRMs. Here, we demonstrate that crystallographic SAR (xSAR) can be extracted from CRMs evaluated *via* HT X-ray crystallography. We developed a simple rule-based ligand scoring scheme that identifies conserved chemical features associated with crystallographic binding and non-binding. Applied to a crystallographic dataset of 957 fragment elaborations in CRMs targeting PHIP(2), a therapeutically relevant bromodomain, our xSAR model demonstrated effectiveness in two proof-of-concept experiments. First, it recovered 26 missed binders in the initial dataset (false negatives), doubling the hit rate and denoising the dataset. Second, it enabled a prospective virtual screen that identified novel hits with informative chemistries and measurable binding affinities. This work establishes a proof-of-concept that xSAR models can be directly extracted from large-scale crystallographic readouts of CRMs, offering a valuable methodology to build SAR models and accelerate design-make-test iterations without requiring CRM hit resynthesis and confirmation. This invites future work to utilise advanced analytics and modelling techniques to further strengthen purification-agnostic workflows.

## Abstract 2

Crystallographic SAR (xSAR) can be extracted from high-throughput crystallography data of crude reaction mixtures, retaining both binders and non-binders to recover missed hits and enable prospective virtual screening.

## Graphical abstract

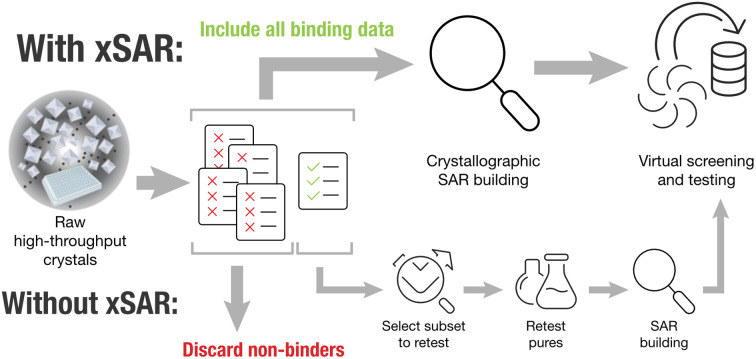

## Introduction

Fragment-based drug design (FBDD) is an effective approach for designing compounds with activity against protein targets.^1,2^ Screening fragments (<18 heavy atoms) usually results in more efficient binding than hits from traditional high-throughput (HT) screening libraries because a higher proportion of atoms in the fragments form productive interactions with the target.^2–5^ Additionally, fragment screening libraries more effectively sample chemical space than HT screening libraries,^2^ resulting in a diverse sampling of scaffolds that can be methodically expanded.

Once the fragment hits have been identified, one aim is to design molecules with higher binding affinity, which often involves growing hits to form interactions with protein residues in the binding site. One approach is to test close analogues of the fragment hits to obtain structure–activity relationship (SAR) studies.^6–8^ SAR studies attempt to build a dataset relating chemical modifications to changes in binding. Although, the SAR landscape can be highly discontinuous where molecules with only a minor chemical difference have very different activities (activity cliffs).^9–11^ Therefore, maximising the sampling of the chemical space is important to understand and use the underlying SAR landscape.

A method to increase the sampling of compounds experimentally tested within a resource limit is to test crude reaction mixtures (CRMs). Testing CRMs has the advantage of bypassing purification, which is a known resource intensive bottleneck.^12^ However, hits identified from the direct evaluation of CRMs such as DNA-encoded libraries,^13,14^ surface plasmon resonance (SPR),^12,15^ and cell-based assays,^16,17^ in so called “Direct-to-Biology” (D2B) approaches, are typically resynthesised and validated. This is because the initial readout can contain false negatives and positives due to various artifacts,^18,19^ including compound aggregation^20,21^ and metal impurities.^22^ For example, Adams *et al.*, screened 92 compounds in CRMs by SPR off-rate screening and selected three compounds to resynthesize, purify, and retest with SPR.^15^ In this work, we asked whether it is possible to extract meaningful SAR directly from CRM readouts without costly and restrictive revalidation, accepting that some false signals will persist, but testing whether predictive models can still be constructed in the presence of this noise. Indeed, prior studies have explicitly characterized both the noise and reproducibility of DEL readouts^23^ and the robustness of SPR applied to CRMs.^12,24^

To explore this possibility, we used HT X-ray crystallography which has shown to be useful in evaluating fragment follow-up compounds in CRMs by measuring structural information for 100s–1000s of compounds.^25,26^ This is enabled by crystal lattice binding sites which selectively capture compounds within the CRM and the associated binding modes can be unambiguously resolved from electron density maps. Although this method is not devoid of false signal, as the starting material might be resolved over the preferred product^27^ or, as is a problem with any X-ray crystallography readout, the wrong ligand might be fit.^28^ Despite these sources of false signal, we hypothesize due to the HT nature of the experiment, the possible individual ligand misassignments are diluted, allowing for robust trends to emerge at the level of shared chemical features rather than single measurements.

Such a large-scale crystallographic dataset of fragment elaborations offers an opportunity to build a SAR model directly from the evaluation of CRMs, precisely because the volume of measurements might compensate for the inevitable experimental noise. While the dataset is non-traditional in a SAR context, since it is binary (hit or no hit) and typically, continuous biochemical or biophysical data are used for SAR studies, crystallographic SAR provides a structural understanding of chemical modifications of a ligand and how that is favoured or unfavoured by the crystal system to result in a binder. Here, we exploit the fact that the dataset is large, so that noisy individual labels can still potentially yield stable, statistically meaningful SAR trends. This opportunity to directly build a SAR model from the positive and negative crystallographic results of CRMs (including the false signal) has not yet been explored.

Here we present a proof-of-concept for developing SAR models directly from HT crystallographic evaluation of fragment elaborations in CRMs, named “crystallographic SAR (xSAR) model”. Our xSAR modelling approach is a simple ligand-based framework that evaluates chemical feature conservation amongst crystallographic binders and non-binders to extract important features and score compounds for predicted binding outcomes. We applied this ligand-based xSAR model to fragment elaborations targeting the second bromodomain of the Pleckstrin-homology domain interacting protein (PHIP(2)), a potential oncology target, following an initial X-ray crystallographic fragment screen. The initial fragment was identified by the SAMPL7 challenge^29^ and selected to inspire further elaborations. The fragment elaborations were experimentally carried out by robotic synthesis where CRMs of 1876 designs were screened by X-ray crystallography on the PHIP(2) target resulting in 22 experimental binders.^30^ From this set of 22 ligands, an initial xSAR model was created using conserved ligand features of binders and non-binders and applied in two ways. First, for validation purposes, the xSAR model was used to recover 26 crystallographic binders not originally identified from the initial evaluation of CRMs (false negatives), doubling the original hit rate and effectively denoising the initial dataset. Then prospectively, the xSAR model was used in a virtual screening exercise that identified novel hits with informative chemistries and up to a 10-fold enhancement in binding affinity with respect to the repurified hit identified from the original evaluation of CRMs.

## Results

Here, we outline the development and application of the crystallographic SAR (xSAR) approach, developed from high-throughput (HT) crystallographic data of fragment elaborations in crude reaction mixtures (CRMs). This novel approach leverages conserved chemical features among crystallographic binders and non-binders to provide actionable insights into SAR directly from crystallographic readouts, showing that, for this PHIP(2) case study, useful SAR can be extracted without additional CRM hit resynthesis for validation. The xSAR model exemplifies how binding data from CRMs can guide both retrospective and prospective analysis of fragment elaborations, guiding follow-up hit identification and enabling the exploration of a broader chemical space. We begin by describing the ligand-based approach for the xSAR model construction. This is followed by an experiment to evaluate the model's ability to recover false negatives, denoising the initial dataset. Finally, we demonstrate the prospective utility of the xSAR model through a virtual screening experiment.

### A simple, ligand-based approach for crystallographic SAR (xSAR) model building

**Fig. 1:**
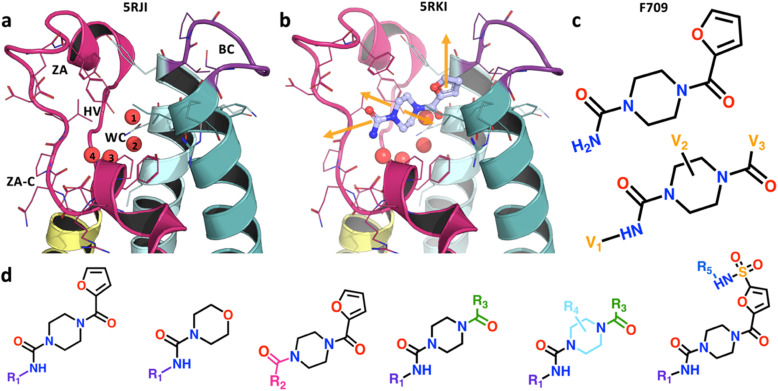
Structural representation of the PHIP(2) binding site, initial fragment hit, and elaboration vectors. (a) The crystal structure of unbound PHIP(2) (PDB ID: 5RJI), in a C2 space group, centred on the acetylated lysine binding pocket with key binding site regions labelled, ZA-loop (ZA), ZA-channel (ZA-C), hydrophobic void (HV), BC-loop (BC), and water cavity (WC). (b) The initial fragment hit, F709 is shown cocrystallised to the pocket (PDB ID: 5RKI) with elaboration vectors (orange arrows) suitable for enhancing interactions with binding site regions. (c) The F709 fragment hit structure with key elaboration vectors (V_1_, V_2_, V_3_) annotated. (d) The six scaffolds of the elaboration series produced by robotic synthesis with the modifications at various R groups highlighted.

In our previous study,^29^ a crystallographic fragment screen for the second bromodomain of the target, PHIP2, resulted in 47 fragments bound to the pharmacologically relevant acetylated lysine (Kac) binding site (Fig. 1a). From the output of the SAMPL7 challenge, a fragment (F709, PDB: 5RKI) was selected with vectors for automated chemistry elaborations (Fig. 1b and c).^30^ Elaborations were produced by automated synthesis routes (as detailed in previous work^30^), resulting in 1876 designs in CRMs screened with X-ray crystallography at XChem,^31,32^ a HT fragment screening facility at Diamond Light Source. The chemistry dictating fragment modification was guided by the availability of building blocks and reactions executable on the robotic platform, ranging from simple one-step syntheses to parallel reaction sequences of up to five steps (Fig. 1d). The reactions were also designed to generate structure–activity relationships. Additionally, deletions were imposed on the parent fragment, and in some cases parts of the scaffold were substituted, such as replacing the piperazine with a larger ring or a methylpiperazine. From a structural perspective, elaboration vectors were selected to sample unoccupied regions of the binding site, including the ZA-channel (ZA-C), hydrophobic void (HV), BC-loop (BC), and water cavity (WC) (Fig. 1a). A full description of the design and chemistry is available in our previous paper.^30^

**Fig. 2:**
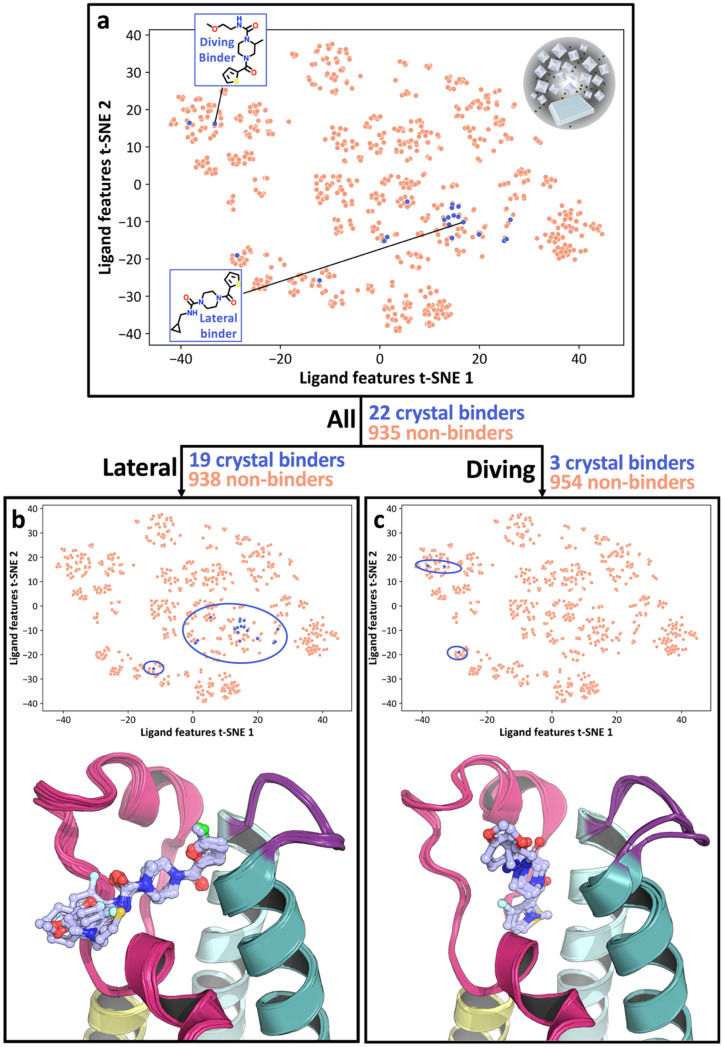
Visualisation of the chemical space and binding outcomes from robotic fragment elaboration *via* HT crystallographic evaluation of CRMs. The three pose datasets are shown as: (a) All poses, (b) the Lateral pose subset, and (c) the Diving pose subset. The binding compounds are coloured blue and circled in (b) and (c). The chemical space defined by the 957 ligands is projected into 2D by t-SNE dimensionality reduction of the fingerprints. The structures in the bottom of (b) and (c) display the crystal binders for the corresponding pose subset.

**Fig. 3:**
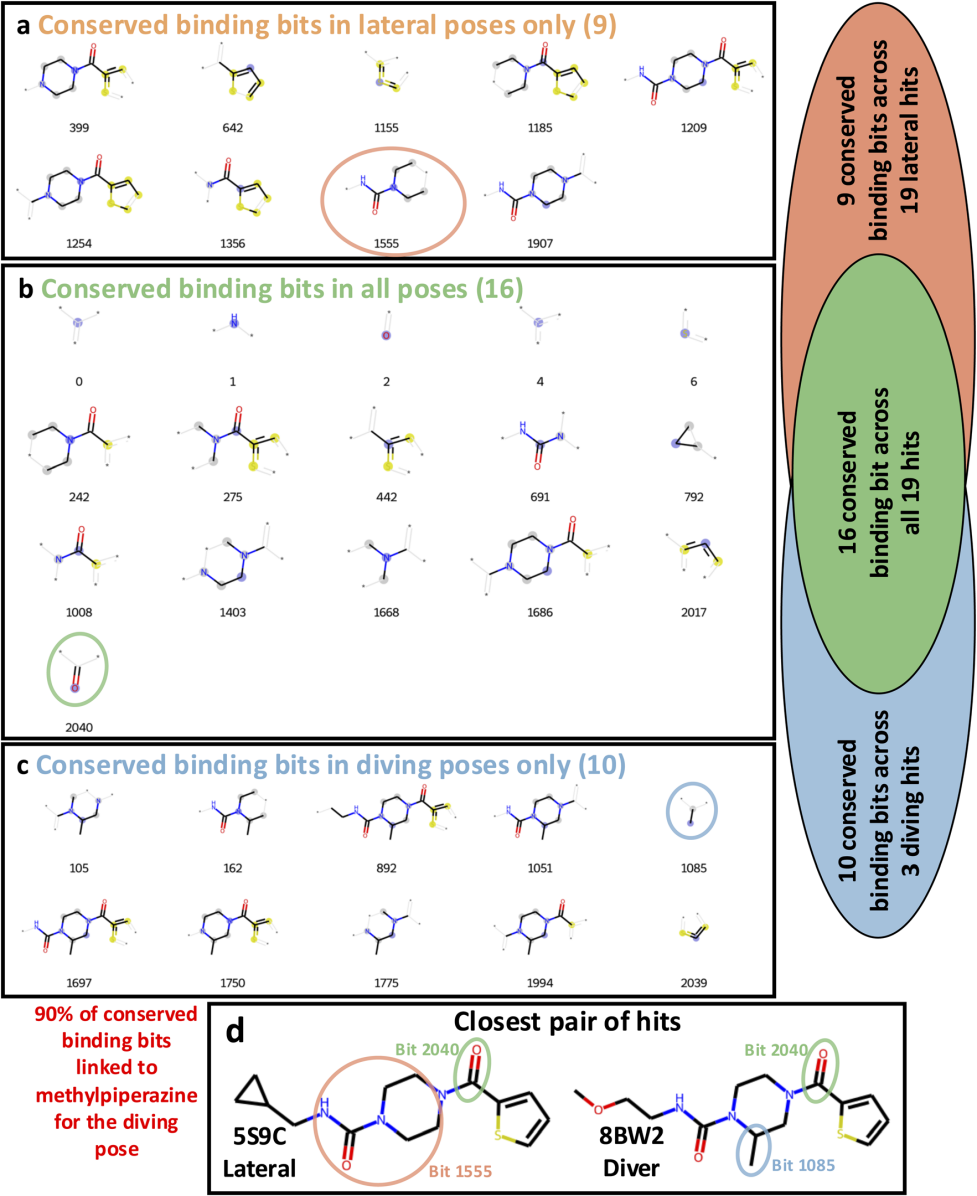
Identification of conserved binding bits (CBB) enables comparison of ligand features driving pose-specific binding. The CBB for (a) Lateral, (b) All, and (c) Diving binders are compared, highlighting pose specific binding features. As indicated by red arrows in (c), 90% of the CBB of the diving binders contain a methylpiperazine moiety. Atoms and bonds belonging to the substructure that turns on the corresponding fingerprint bit are drawn with bold bonds; atoms in that substructure are colour-coded, with the source atom highlighted in blue, aromatic atoms in yellow, and aliphatic atoms in grey. Lighter bonds and atoms marked with an asterisk are not part of the bit but are shown to provide visual context. The Venn diagram illustrates pose-specific and pose-unspecific bit ensembles: CBB only of the diving pose are shown in blue, CBB across all poses are shown in green, and CBB only of the lateral poses are shown in orange. This shows how there are bits within each of the CBB sets that are only found in pose-specific ensembles and are also pose-unspecific. (d) Shows the most chemically similar pair of crystallographic hits with varying poses, along with PDB codes, illustrating how CBB map onto actual molecules.

The crystallographic outputs from the evaluation of CRMs were curated according to (1) successful synthesis outcomes, as measured by automated Liquid chromatography-mass spectrometry (LC-MS) analysis of CRMs, and (2) high quality X-ray diffraction data, leading to a final subset of 957 fragment elaborations (Fig. 1c and d) hereafter referred to as the OriginalRefined-957 dataset (Fig. 2). This implies that only data points with measurable quality control traces and usable electron densities were considered. All ligands were labelled with a binary value based on their crystallographic outcome where “binder” was defined as a compound resolved from the electron density, and “non-binder” lacked crystallographic evidence of binding. To help understand the spread of chemical space explored by the ligands, t-Distributed Stochastic Neighbourhood Embedding (t-SNE) based dimensionality mapping was employed, seen in Fig. 2. Morgan Fingerprints^33^ were input into t-SNE. In this scheme, RDKit enumerates atomic environments around atoms and encodes their connectivity invariants (atomic number, valence, formal charge, aromaticity, ring membership, *etc.*), setting the corresponding binary “bits” when those substructures are present. These fingerprints therefore represent local atom–bond environments as binary features capturing the presence or absence of specific chemical motifs, within a given molecule, and were used as input to t-SNE (Fig. 3).

The OriginalRefined-957 dataset was then divided into three data subsets based on pose information, “All”, “Lateral” and “Diving”. The All set aggregates hits from both poses (Fig. 2a). The lateral pose corresponds to fragment F709 follow-up compounds (Fig. 1) binding in a conserved orientation to the original fragment, covering the binding site laterally hence not interacting with the water cavity (Fig. 1a and 2b).^30^ The diving pose corresponds to fragment F709 follow-up compounds (Fig. 1) binding in an alternative orientation, involving a 90° rotation around the core piperazine ring compared to the lateral pose and occurs with displacement of water molecules composing the water cavity (Fig. 1a and 2c).^30^ In our dataset splits, diving poses are counted as non-binders in the Lateral split and lateral poses are counted as non-binders in the Diving split. A more detailed structural rationale for the observed lateral-to-diving pose transition is available in our previous work.^30^ Overall, the addition of a methyl substituent on the piperazine appears to induce the rearrangement by providing apparent favourable hydrophobic contacts within the binding site while simultaneously destabilising the lateral orientation. In addition, computational analysis indicated that the PHIP(2) water network is relatively unstable,^34^ so its displacement may further enable the diving pose by releasing water molecules into solvent leading to an entropic gain. However, we do not have direct thermodynamic data for these PHIP(2) crystallographic ligands, so this should be regarded as a qualitative hypothesis that would require dedicated experiments to test. This can be achieved *via* a combination of isothermal titration calorimetry,^35^ crystallography and simulations^36^ which extends beyond the scope of this study.

Most lateral binders cluster together in the central region of the chemical space, as seen in the central blue circle in Fig. 2b, reflecting similar chemical features for those compounds. In contrast, diving binders are more dispersed, with two hits in the top-left and one hit in the bottom-left clusters of the chemical space (Fig. 2c). This dispersion highlights the difficulty, here, of capturing subtle chemical influences on binding outcome and pose. Specifically, the methyl addition to the piperazine ring, a small chemical modification, was hypothetised to drive the switch from lateral to diving poses (Fig. 3).^30^ Although direct matched pairs were not previously resolved, two compounds with high chemical similarity were observed in the lateral and diving poses, seen in Fig. 3d, with the methyl addition appearing to be the most relevant difference, as indicated by electron density. However, a single methyl group change did not create a distinct clustering pattern highlighting that capturing the nuanced relationship between small chemical modifications and xSAR may require a different approach (Fig. 2). Some clusters also completely lack crystallographic binders, such as the centre-bottom cluster in Fig. 2a, which is populated with sulphonamide-containing compounds seen as one elaboration series in Fig. 1d. Similarly, the absence of binders for this series presents an opportunity to identify chemical features associated with non-binding behaviour. These observations suggested that a ligand-based feature method could recover chemical features associated with binding and non-binding crystallographic readouts of fragment elaborations.

To address this, we created a crystallographic-SAR (xSAR) model, where activity, or lack thereof, was defined as the binary crystallographic binding outcome and is used to rationalise pose-specific crystallographic binding of a given compound based on conserved binding and non-binding features. To build the xSAR model, binding and non-binding features (hereafter referred to as bits) were extracted from the Morgan fingerprints^37^ of the compounds in the OriginalRefined-957 dataset. Conserved binding bits (CBB) were those found in all binding compounds, seen in Fig. 3 for each pose set. Conserved non-binding bits (CNB) were bits only found within the non-binding compounds. We identified these bits for the All, Lateral, and Diving datasets (Fig. S1) to isolate pose-specific conserved bits (Lateral or Diving) and have a more general metric where all poses are considered (All). The association between the methylpiperazine modification and the diving pose can be seen as this moiety is present in 90% of the diving pose specific CBB (Fig. 3c).

**Fig. 4:**
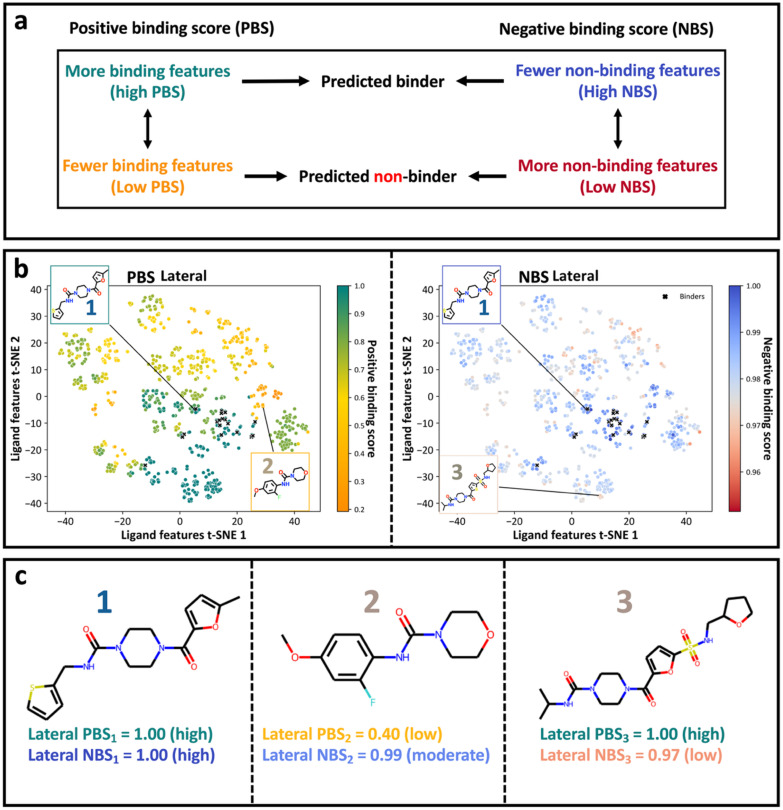
xSAR landscapes quantified by the Positive Binding Score (PBS) and Negative Binding Score (NBS) metrics reveal binding trends within the robotically defined chemical space. (a) Schematic representation of the PBS and NBS and their link to propensity of binding. A high PBS indicates the presence of conserved binding bits, whereas a high NBS indicates the absence of non-binding bits. Conversely, a low NBS indicates the compound contains features associated with non-binding, suggesting a non-binder. (b) Visualisation of the chemical space projected onto 2D from t-SNE dimensionality reduction of the fingerprints. The left panel displays the PBS landscape (coloured from low (orange) to high (green) PBS), and the right panel displays the NBS landscape (coloured from low (red) to high (blue) NBS). Crystallographic binders from the OriginalRefined-957 dataset are indicated with black crosses. (c) Chemical structures of highlighted compounds, with their corresponding PBS and NBS values for the lateral pose. Compound 1 has a maximum PBS and high NBS, making it a strong binder candidate. Compound 2 has low a PBS and moderate NBS, indicating weaker binding features. Compound 3 has high a PBS but lower NBS, suggesting the presence of both binding and some non-binding characteristics. The numbering of the molecules in panel b relates to the molecules displayed in panel c.

We then used the CBB and CNB sets to calculate the Positive Binding Score (PBS) (eqn (2)) and Negative Binding Score (NBS) (eqn (3)) for individual compounds. These scores quantify the recovery rate of conserved binding and non-binding bits for a given compound and are defined in the Methods. The PBS of a compound is the number of activated bits in the compound that are found in the CBB set, divided by the total CBB count. A PBS of 1 indicates that a compound recovers all conserved binding bits. The NBS of a compound is the complement of the number of activated bits in the compound that are found in the CNB set, divided by the total CNB count. A NBS of 1 indicates that a compound does not recover any conserved non-binding bits. A compound with both high PBS and NBS values should in theory have a greater propensity of binding, as it contains many conserved binding bits while lacking non-binding bits (Fig. 4a). The exact definitions for these categorisations can be seen in the method whilst the computational implementation, with exemplar calculations, are available in the GitHub repository.

The chemical space of the OriginalRefined-957 dataset projected into 2D by t-SNE dimensionality reduction of the fingerprints and coloured by PBS or NBS values is visualized in Fig. 4b, also called the xSAR landscape. The PBS and NBS values were calculated using the All, Lateral and Diving bit sets resulting in three sets of two scores or six xSAR landscapes in total. The analysis presented here is for the Lateral bit set and the full analysis for all three datasets is shown in Fig. S2. These xSAR landscapes provides a quantitative method to link ligand features to crystallographic binding outcomes for the chemical space defined in the HT evaluation of robotically generated CRMs.

In Fig. 4b, the PBS and NBS landscapes are overall in agreement, especially where both scores are high. The lower right cluster seen at the landscape coordinates of Ligand features t-SNE 1: 20 and Ligand features t-SNE 2: −10 shows the experimental binders and similar compounds where most have PBS and NBS scores around one. However, some clusters can show divergent PBS and NBS scores, such as the cluster around landscape coordinates of ligand features t-SNE 1: 30 and Ligand features t-SNE 2: 0, where data points have low PBSs and high NBSs. This is due to the absence of the amide and 5-membered ring essential for binding whilst not bearing any groups associated with non-binding (Fig. 3). Reversibly, compounds with a high PBS and slightly low NBS are seen near landscape coordinates of Ligand features t-SNE 1: −30 and Ligand features t-SNE 2: 5. These sulphonamide compounds (Fig. 1d) were all part of the same elaboration series containing an extension at the furane ring which repeatedly led to non-binding while also containing many conserved binding bits (Fig. 3). This illustrates the ability of this approach at mapping positive and negative crystallographic SAR.

The range of PBS and NBS values differs significantly. PBS ranges from 0.20 to 1.00, while NBS is much more constrained, ranging from 0.95 to 1.00. This shallower NBS gradient arises from the larger total CNB count, as non-binders outnumber binders (935 *vs.* 22). Additionally, the congeneric nature of those elaborations implies a relatively focused chemical space, leading to lower structural diversity across the dataset. As a result, fewer unique bits are activated throughout the dataset, increasing the proportion of CNB. Consequently, NBS values tend to be higher than PBS (Fig. S4).

This xSAR method offers a simple yet rigorous quantitative approach for extracting binding and non-binding signal from HT crystallographic readouts, using ligand features. In the following sections, we evaluate the predictive power of these PBS and NBS metrics through both retrospective and prospective tests, which will help establish their practical utility.

### xSAR significantly recovers false negatives from initial crystallographic evaluation of CRMs

Visual inspection of the individual datasets revealed compounds initially identified to be non-binding with PBS and NBS of 1 (Fig. S3) indicating that may well be binders (false negatives). In the context of HT crystallographic evaluation of CRMs, there is an increased combination of experimental factors that increase the risk of not measuring binding compounds, such as defective quality control for synthetic reactions, poor relative crystal tolerance to ligands in CRMs, low compound solubility-concentration,^38^ inaccurate dispensing, and as well as crystal pathologies.^25,29,39,40^ It is also possible that CRMs contain unstable or reactive products, side products, implying that degradation between quality control (QC) analysis and crystallographic evaluation may have taken place.^28^

Given the potential presence of false negatives, we decided to test if the xSAR model could identify false negatives from the OriginalRefined-957 dataset. We selected and tested a subset of the OriginalRefined-957 dataset based on budget constraints and compound availability in pure form from Enamine (Fig. S5). We applied log sampling across PBS and NBS metrics for all three pose datasets (All, Diving, Lateral) with increased sampling density at higher scores (see Methods for the exact resampling procedure). This process yielded 97 compounds, which comprise our Retrospective-97 set. Each compound in this set was evaluated using all six possible scoring schemes regardless of which ensemble originally selected it (Fig. 4).

**Fig. 5:**
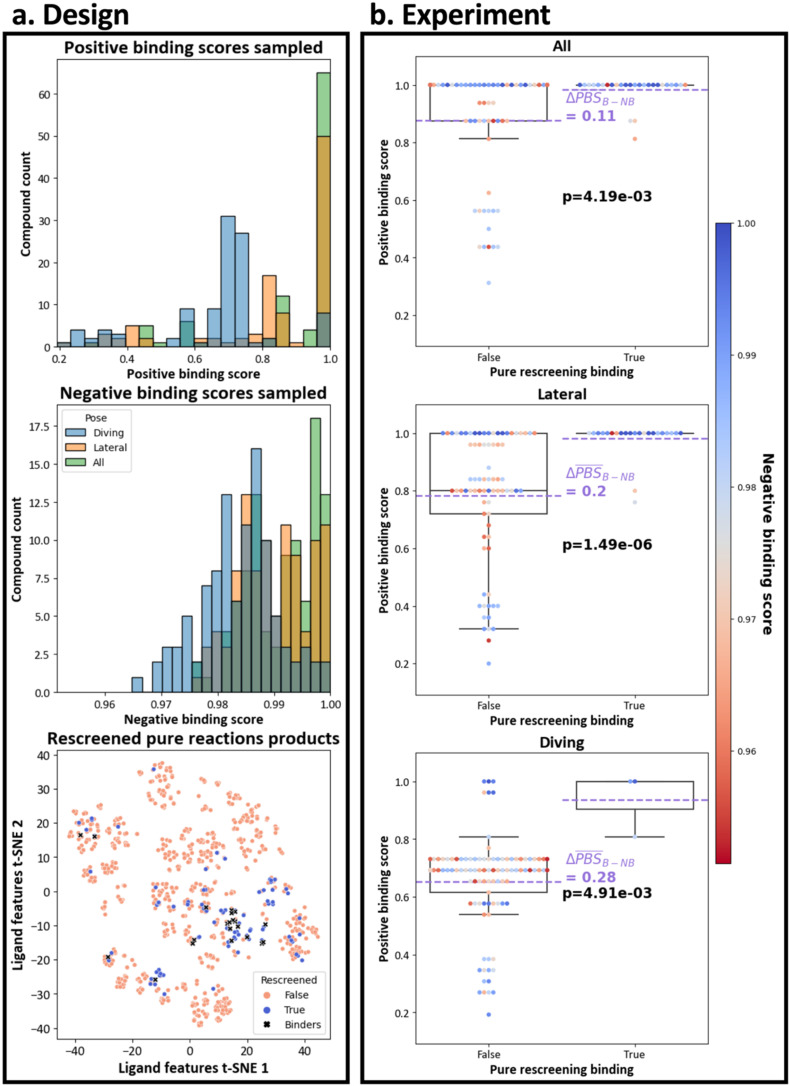
The PBS significantly enriches false negative identification in crystallographic re-evaluation of pure fragment follow-up compounds. The left panel (a) shows the distribution of PBS and NBS values of the Retrospective-97 set as histograms on the top. The 2D t-SNE dimensionality reduced plot of the fingerprints of the OriginalRefined-957 dataset is shown on the bottom, coloured by presence in the Retrospective-97 set (blue) and binding compounds represented by black crosses. The right panel (b) presents the distribution of PBS values of the Retrospective-97 set in box plots depending on the pose set and grouped into the true or false crystallographic rescreening binding outcome. The points are coloured by NBS value (from low (red) to high (blue)). Purple dotted lines show the average PBS values for each class and the resulting difference (ΔPBS) between binders (True) and non-binders (False). The *p*-values (in bold) from the Mann–Whitney *U* tests, indicate the significance of the PBS in discriminating binders from non-binders.

The selection process yielded varying score distributions for the 97 compounds depending on which pose-based data subset they were calculated from (Fig. 5a). The All set had score distributions shifted to high values for both PBS and NBS because the underlying conserved positive and negative binding bits are less specific, for example aliphatic and aromatic carbons (Fig. 3), and more likely to be activated in selected compounds, overlapping between the Lateral and Diving subsets (Fig. S4). This implies that the log sampling strategy for the Lateral and Diving data subsets also selected for high-scoring compounds for the All set. The Lateral subset had the second most shifted distribution to high values for both scores because more compounds with Lateral-like features were generated in the initial robotic synthesis (Fig. 5a). Conversely, few compounds had a methylpiperazine group, resulting in the least high value shifted score distribution for the Diving subset. As shown in the 2D t-SNE plot in Fig. 5a, the sampled compounds were drawn from across the chemical space of the OriginalRefined-957 set with a focus around the clusters of known binders as expected since higher PBS and NBS values were prioritised. The selection was also partially limited due to the availability of compounds (Fig. S5) but the score distributions for selected compounds (Fig. 5a) is similar to the distributions for the OriginalRefined-957 set (Fig. S4). The large top-right cluster was not sampled since these compounds had low PBS and NBS values due to the replacement of the essential 5-membered di-heterocyclic ring with various groups and are therefore not expected to bind (Fig. 1d).

The Retrospective-97 compound set was purchased in pure form from Enamine and evaluated with X-ray crystallography, resulting in 26 novel binders at the Kac binding site (23 Lateral and three Diving). This doubled the initial hit rates for both poses: Lateral increased from 1.99% (19/957) to 4.39% (42/957), and Diving from 0.31% (3/957) to 0.63% (6/957) (Fig. 5 and S6). Therefore, the xSAR model could successfully identify false negatives, effectively denoising the outcome of the initial experiment.

In one instance, a hit was resolved outside the Kac binding site, at a site located between α-helices Z and C which has been previously described during the SAMPL7 challenge^29^ where seven fragments were resolved there (PDBs: 5RJJ, 5RJK, 5RJL, 5RJQ, 5RKR, 5RKV, 5RKX) (Fig. S8).

The PBS metrics can recover binders over non-binders with statistical significance. The PBS values calculated using the All, Lateral and Diving binding bits discriminate between true (binding) and false (non-binding) crystallographic evaluation of the Retrospective-97 set (*p*-values below 0.05 from the Mann–Whitney *U* Test). The difference in average PBS between true and false binding outcomes was largest for Diving binding bits (0.28 PBS), compared to Lateral (0.20 PBS) and All (0.11 PBS) (Fig. 5b). This larger difference for Diving binding bits stems from the methylpiperazine moiety being present in 90% of conserved Diving binding bits, while only 11.3% (11/97) of compounds in the Retrospective-97 set contain this feature. Since most non-binding compounds lack the methylpiperazine, their Diving PBS scores are correspondingly lower (Fig. 3). Comparatively, 65 compounds contained a piperazine and 21 had neither piperazine nor methylpiperazine moieties in the Retrospective-97 set.

The PBS and NBS metrics were benchmarked against other common ligand-based classification methods (Fig. S11) on the Retrospective-97 set, with results showing that the PBS metric outperformed the NBS and Tanimoto similarity metrics.^41^ For comprehensive comparison, we included a random forest classifier, which is widely used in ligand-based prediction tasks including bio-availability, bioactivity and toxicity^42–44^ (see SI Methods). We also evaluated two Tanimoto similarity scoring approaches^41,45–47^ that calculated either the mean or maximum Tanimoto coefficient value of a test Retrospective-97 compound against known binding compounds in the OriginalRefined-957 set. Our benchmark revealed that PBS outperformed the random forest classifier on the Diving bit set (PR-AUC: 0.56 *vs.* 0.17). The random forest's poor performance likely reflects the highly unbalanced Retrospective-97 dataset, which contains only 3.09% (3/97) binders *versus* 96.91% (94/97) non-binders for the Diving set, a challenging scenario for this classifier (Fig. S10 and S11). In contrast, the PBS achieved PR-AUC values comparable to the random forest for the All and Lateral bit sets while being an order of magnitude faster to compute (Fig. S12). The random forest's hyper parameters were also optimised against for that task (trained on the OriginalRefined-957 with Retrospective-97 datapoints removed and tested on the Retrospective-97) likely leading to better performance than in a blinded scenario (see SI Methods). In contrast, the Tanimoto-based metrics consistently showed weaker performance across all evaluated bit sets (Fig. S11) hence also demonstrating that our scoring schemes behaves differently than these methods.

**Fig. 6:**
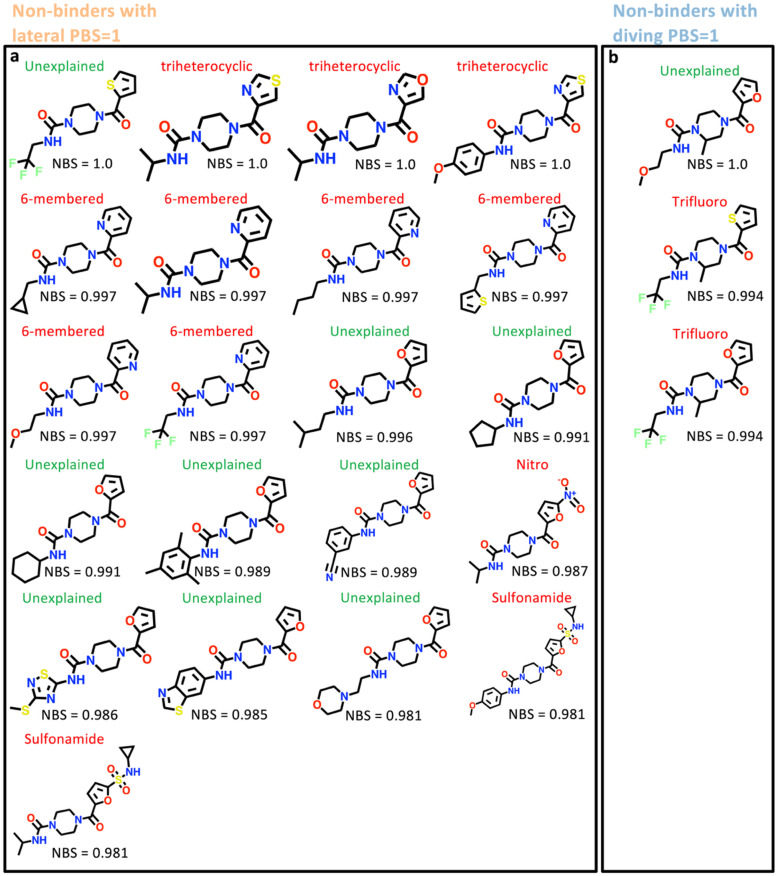
Crystallographic rescreening of pure compounds further identifies features leading to non-binding. (a) Non-binders with a lateral PBS of 1 are boxed in orange and (b) non-binders with a diving PBS of 1 are boxed in blue. Each compound is labelled with features hypothesized to cause non-binding and NBS values. In some instances, compounds with maximum PBS and near maximum NBS have no obvious non-binding features.

Within the Retrospective-97 set, there were 21 and three non-binders with maximal PBS for the lateral and diving poses, respectively (Fig. 6). Features such as five-membered tri-heterocyclic rings, six-membered rings, nitro and sulphonamide groups for the lateral pose, and trifluoromethyl for the diving pose, repeatedly led to non-binding events. By updating these features in the appropriate binding data subset for future PBS and NBS calculations, this would further refine the xSAR model (Fig. 3). Additionally, there were 10 non-binders that had no non-binding features (maximal NBS) that could explain the lack of crystallographic binding (nine scored for the lateral pose and one scored for the diving pose) (Fig. 6 and S7). Possible explanations of not resolving these compounds could include unpredictable mechanical failures, data processing errors^25^ or degradation of crystals in soaking conditions.^29^ Indeed there is even disagreement between repeats of screening pure compounds.^29^ Solubility limitations in ethylene glycol could also lead to aggregation and reduced effective compound concentration.^48^ Here ethylene glycol was used as solvent, instead of DMSO as DMSO has intrinsic affinity for bromodomains.^49^ Crystal conditions can likewise affect solubility; high-salt mother liquors are known to promote precipitation of less-soluble, hydrophobic fragments during soaking, thereby lowering the effective ligand concentration.^50^ In addition, kinetic or diffusion factors may play a role, where binding events occur too slowly to be captured under our soaking protocol. Longer soaking or higher-temperature conditions might increase hit rates, although this may be limited by crystal fragility.^48^

Our xSAR model was able to double the initial hit rates for both poses (Fig. 5) and provides easily interpretable metrics for explaining most non-binders. Altogether, our scoring method is tractable and easily updateable with new measurements, adding to the toolkit of SAR approaches.

### Prospective usage of the xSAR model *via* virtual screening identifies more potent and informative binders

We next use our xSAR model prospectively in a drug discovery scenario and explore a chemical space of commercially available compounds. This experiment was carried out simultaneously with the retrospective study. Hence, the xSAR model used in this experiment was the one directly extracted from the OriginalRefined-957 set (see Data availability).

**Fig. 7:**
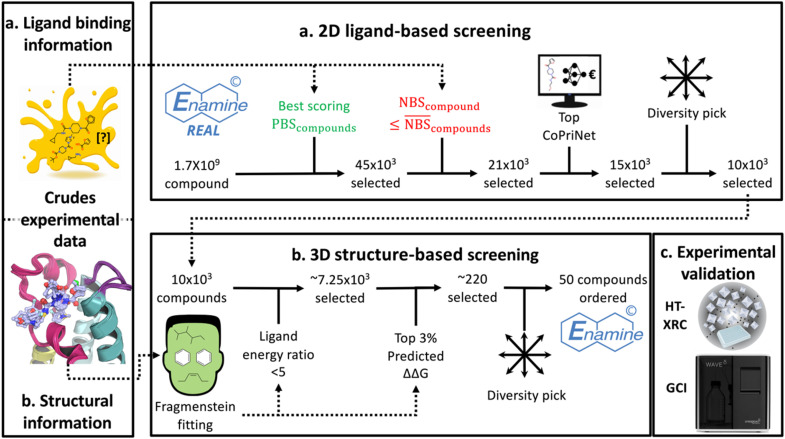
xSAR binding scores and structures measured from initial crystallographic evaluation of CRMs enable integrative virtual screening. (a) First, a ligand-based virtual screening of the Enamine REAL© database was performed using the PBS and NBS metrics. (b) Then, a 3D structure-based screening was performed using predicted conformations and energy values carried out by the tool Fragmenstein.^58^ Finally, the selected set, Prospective-93, totalled 93 compounds (47 scored by the lateral pose and 46 scored by the diving pose) for (c) experimental evaluation by HT X-ray crystallography (XRC) and Grating-coupled interferometry (GCI).

A virtual screening procedure of the Enamine REAL© database^51,52^ was performed resulting in the purchase of pure lead-like compounds^53,54^ that were tested as described in the Methods. First 2D ligand-based filters were applied utilising the PBS and NBS xSAR binding scores. Then binding poses for selected compounds were computed, using the previously resolved high resolution structures, to further filter based on predicted energy values using the Rosetta All-Atom Energy Function^55^ (Fig. 7). The final selection included 47 compounds for the lateral pose (Fig. S13) and 46 for the diving pose (Fig. S14), totalling 93 compounds (hereafter referred to as the Prospective-93 set) bought from Enamine in pure form for crystallographic and kinetic evaluation at XChem. The kinetic evaluation was performed using a grated-coupled interferometry (GCI) assay^56,57^ that evaluates mass accumulation on a biosensor chip similarly to surface plasmon resonance (SPR). Only the Prospective-93 compounds purchased in pure form were tested by GCI and crystallography whereas, the Retrospective-97 compounds were evaluated, solely by crystallography in pure form.

**Fig. 8:**
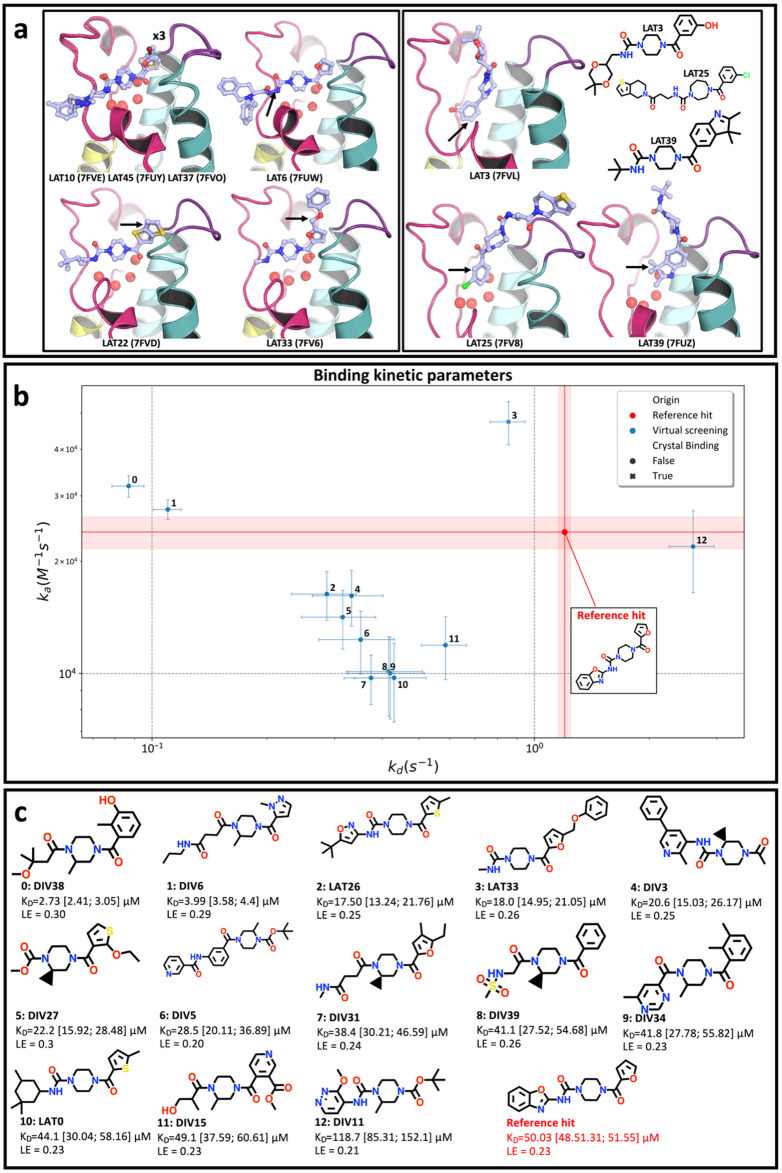
Experimental validation of selected virtual screening compounds reveals informative poses and more potent hits. The top panel (a) shows the results from the crystallographic evaluation of virtual screening compounds (Prospective-93). Overall, nine compounds were resolved, where six maintained the initial lateral pose (left panel). Among the lateral binders, three displayed novel features (left panel with black arrows pointing to the features), whereas three exhibited modified binding (right panel) compared to the lateral pose. The middle panel (b) shows results for the kinetic evaluation of virtual screening compounds with GCI where experimentally determined dissociation rate (*k*_d_) and association rate (*k*_a_) values are showed with associated errors, in s^−1^ and, M^−1^ s^−1^, respectively. The reference binder, resolved from CRMs in the previous work,^30^ is represented by a red dot and shaded regions indicating *k*_a_ and *k*_d_ values and errors. GCI hits resolved in crystals are marked by points and unresolved hits are marked by crosses. The 2D structures for each GCI hit are shown on the bottom panel (c), ordered by increasing dissociation rate (*K*_D_) (upper and lower error ranges calculated from *k*_a_ and *k*_d_ errors noted in parentheses) with integer identifiers corresponding to the kinetic parameters scatterplot (b). The ligand efficiency is also noted for each compound using the number of non-hydrogen heavy atoms.

Crystallographic evaluation of the pure catalogue compounds yielded nine binders, achieving a hit rate of 9.68% (9/93) (Fig. 8a). Only compounds scored using the lateral pose-specific PBS and NBS were crystal binders, resulting in hit rates of 19.15% (9/47) and 0.0% (0/46) for the lateral and diving poses, respectively. Although not all bound compounds had a lateral pose, six had a conserved lateral pose while three did not (Fig. 8a). Among the six binders with a conserved lateral pose, three had a similar molecular structure to previously resolved compounds, while the other three presented novel groups. Seen in Fig. 8a of the binders with the lateral pose conserved, one binder had a 1,2-diformylhydrazine bond forming new hydrogen bonds (7FUW, LAT6), another had an unobserved bicyclic thieno[3,2-*b*]thiophene ring system that made amplified hydrophobic contacts with neighbouring amino acids (7FVD, LAT22), and the third had an anisole extension to the furan that bound in a previously unproductive (*i.e.* sulfonamide extensions) vector (7FV6, LAT33). The remaining three binders exhibited modified conformations with respect to the lateral pose used for template fitting, including one with a phenol ring that displaced water molecules, similarly, to diving compounds (Fig. 2), with an unaltered piperazine ring occupying the central cavity (7FVL, LAT3). Two compounds flipped and translated without displacing the water network and had long extensions towards the solvent (7FV8, LAT25 and 7FUZ, LAT39) (Fig. 8a).

The absence of crystal hits scored by the diving binding pose (0.0% hit rate) highlights a suspected limitation for the Diving PBS and NBS metrics' ability to distinguish between binding and non-binding compounds, during virtual screening. This low hit rate compared to the lateral pose hit rate (12.77% (6/47)) may stem from the limited chemical diversity and number of compounds associated with the diving pose *versus* lateral pose in the OriginalRefined-957 set (3 *vs.* 19 binders). In the OriginalRefined-957 set, most diving compounds contained a methylpiperazine moiety (Fig. 3). This feature likely influenced the adoption of the diving pose. However, the small number of confirmed binders limits confidence in the conserved binding features identified for this pose. In addition, the relatively sparse sampling of methylpiperazine-containing compounds likely introduces uncertainty into the resulting scores. Hence, broadening the underlying sampling, in future experiments, could help enhance the resolution of pose-specific metrics in future iterations.

GCI kinetic measurements identified 13 binding events (Fig. S15) based on the hit-calling criteria (*k*_a_ error < 25% and *k*_d_ error < 25% and *R*_max_ > 10), demonstrating an application of using xSAR to identify binding compounds from a distinct chemical space. While there is no universally accepted standard for kinetic hit calling, we applied more stringent cutoffs than reported elsewhere to ensure as robust as possible hits were selected.^59^ Overall, 61.54% (8/13) of these new hits showed an apparent increased ligand efficiency (LE) compared to the reference compound, with 92.31% (12/13) also indicating an improved *K*_D_, which further exemplify the usage of the xSAR-based virtual screening to prioritise compounds for kinetic validation.

As seen in SI Fig. 15, the absolute response levels and fitted *R*_max_ values may vary substantially between ligands. For an ideal 1 : 1 interaction, the theoretical *R*_max_ on a given surface is set mainly by the amount of immobilised receptor, its molecular weight (here ∼17,6 KDa), and the analyte molecular weight (and refractive index).^60^ Using the PHIP(2) loading (6554 pg mm^−2^) and ligand molecular weights, the expected *R*_max_ values are ∼130.1 pg mm^−2^ for DIV6 and ∼127.9 pg mm^−2^ for LAT33. The fitted *R*_max_ for DIV6 is at about 99% its theoretical *R*_max_, whereas LAT33 reaches only 9% of its theoretical *R*_max_, indicating that the >10-fold difference arises from a lower fraction of binding-competent PHIP(2) for LAT33. Because all ligands were injected back-to-back on the same chip in a rapid screening mode, fitted *R*_max_ values can also capture non-idealities such as baseline drifts, variations in bulk refractive index/DMSO content, protein degradation and non-specific binding. The apparent steady-state plateau observed for LAT33 at the highest concentration may reflect equilibrium binding to this smaller active subpopulation rather than saturation of the entire immobilised protein layer, consistent with the possible impact of random amine coupling, orientation-dependent epitope accessibility^61^ and gradual loss of activity during extended back-to-back injections on the fraction of active ligand.^62^ Here, we use *R*_max_ primarily as a qualitative QC metric, to remove binding events with low signal, and base our comparisons using *k*_a_, *k*_d_, *K*_D_ and ligand efficiency. More elaborate kinetic triaging or alternative models would likely refine individual fits but were beyond the scope of this fast, high-throughput experiment.

Nevertheless, Two compounds (DIV38 and DIV6 in Fig. 8c) showed an approximate order of magnitude improvement in *K*_D_ compared to the original assay binder identified previously,^30^ seen as the red labelled compound in Fig. 8b. Additionally, the LE of the best new hits, 0.30 and 0.29, appeared increased compared to the LE of the original assay binder, 0.23.^30^ This indicates that these compounds make more productive interactions with the target, rather than contributing affinity through nonspecific hydrophobic bulk.^63^ There is an opposite trend of pose-specific hit rates between the GCI assay compared to crystallography hits. The pose-specific hit rates from the GCI assay are 6.38% (3/47) and 21.74% (10/46) for compounds selected from lateral and diving scored sets (Fig. S13–S15). Whereas there are more lateral binders and no diving binders from crystallography. Most of the GCI hits (76.92%, 10/13) contained a methylpiperazine ring which was a feature initially associated with the diving pose and known to be resolved in crystals at a lower rate than lateral poses (Fig. 8a). Binder three (7FV6, LAT33) was selected from the lateral scored set and was a hit in both crystals and the GCI assay. To visualise where the prospective compounds lie relative to the robot-defined chemical space, we recomputed a t-SNE map including the OriginalRefined-957 together with the Prospective-93 (SI Fig. 16). Although absolute positions differ from Fig. 2 (different dataset and stochastic embedding), the lateral-scored prospective compounds cluster near the region that contains the known lateral crystallographic binders, whereas the diving-scored prospective compounds occupy a more peripheral region. Consistent with the experimental outcomes, GCI hits are enriched among the diving-scored compounds, and the diving-scored GCI hits themselves cluster together, indicating a kinetically active and distinct chemotype within this region.

Overall, we exemplified how the xSAR model can be applied in a prospective manner, integrating both ligand- and structure-based information, resulting in nine compounds resolved in crystal structures, along with poses and previously unresolved chemical groups. In the GCI assay, out of 13 binding events, two hits were identified with a measured >10 times improvement in *K*_D_ compared to the original assay binder.^30^ With the acquired structural information, further optimisation of binding affinity can be pursued using structure-based computational methods. However, full kinetic profiling and orthogonal assays of these GCI hits, would be required for detailed characterisation and confirmation, before optimisation or SAR driven decision making, but was beyond the scope of this proof-of-concept. The aim of this experiment was to show how the xSAR model can be used to rapidly select catalogue compounds.

## Discussion

Here, we introduce a simple methodology for directly extracting a tractable xSAR model from a HT crystallographic experiment of fragment elaboration series in robotically generated CRMs (Fig. 2) using a ligand-based framework (Fig. 4).

The PBS and NBS, used in the xSAR model offer complementary insights into the binding propensity of compounds by quantifying the presence of conserved binding and non-binding features, respectively (Fig. 4). A high PBS indicates that a compound shares many features with known binders and therefore has a higher propensity to exhibit a particular binding pose. Conversely, the NBS captures the extent to which a compound contains features associated with non-binding. A high NBS indicates that a compound does not contain these non-binding features, increasing the potential of it being a true binder. Our xSAR approach differs from, and outperforms, classical Tanimoto-based similarity metrics at recovering false negatives (Fig. S11).

In our experiments, the PBS was more effective than the NBS in discriminating binders from non-binders (Fig. 5, S7 and S11). However, the NBS can identify compounds with features repeatedly leading to non-binding, providing useful and complementary SAR information (Fig. 4). The lower performance of the NBS compared to the PBS is likely due to the difficulty in categorising non-binding bits as they address an unobserved signal with multiple and potentially additive causes including solubility, thermodynamics, protonation and other chemical and physical factors affecting binding outcomes that our method is agnostic of.^64^ Additionally, the greater number of non-binding features resulted in a narrower NBS range (minimum 0.95) compared to PBS (minimum 0.20) for the OriginalRefined-957, possibly limiting its discriminatory power. Although non-binding features are more numerous in total (Fig. S1), each individual non-binding feature appears infrequently across compounds, this creates a diffuse negative class with greater variability attributing to the challenging classification problem. These limitations suggest potential for advanced analytics, such as consensus scoring schemes^65^ that account for both binding and non-binding features together rather than separately, or machine learning approaches although dataset size and imbalance may be limiting here (Fig. S10). Leveraging negative binding outcomes is routine for classifying active *versus* inactive compounds for solution assays,^66,67^ but has yet to be explored in the crystallographic context. In this work, while not only was extracting a SAR model from HT crystallographic data novel, additionally new was scoring the negative binding outcomes into a separate score, NBS.

After establishing the PBS and NBS as metrics for evaluating binding potential, we applied these scores to analyse the OriginalRefined-957 set (Fig. 5). This analysis revealed several important findings, particularly related to the identification of 26 false negatives (instances that were a positive readout within the Retrospective-97 dataset but resulted in an initial negative readout in the OriginalRefined-957 dataset, Fig. S9). Although the compounds selected in this study from the initial evaluation of CRMs by X-ray crystallography had positive QC traces and interpretable electron density (*i.e.* a diffraction dataset that can be successfully processed for PanDDA analysis^68^), some still failed to yield crystallographic complexes. Given these positive quality-control gates and the multiple possible sources of crystallographic noise, it is difficult to rationalise the precise origin of each false negative. Our focus here was not to resolve these causes exhaustively, but to demonstrate that false negatives exist and can be systematically recovered through xSAR-guided follow-up experiments. Determining their specific origins would require more advanced analytical studies, which lies beyond the scope of this proof-of-concept.

With any classification procedure, especially on the HT level, there will always be misclassification due to noisy data. Noise analysis is already being addressed in DNA-encoded library approaches, for example, Satz found that activity readout patterns arose from formation of truncates rather than true activity.^69^ Other X-ray studies of CRMs did not, to the best of our knowledge, address false negatives in the context of SAR model building, and likely have missed interesting binders,^70^ although their aim may have been to rapidly generate data. Overall, our approach has proven effective in retrospectively identifying false negatives, effectively denoising the initial CRM readout (Fig. 2) through follow-up experiments (Fig. 5) resulting in a more thorough xSAR model. The recovery of false negatives highlights the intrinsic noise of HT crystallographic evaluation of CRMs. Although this does not directly accelerate the identification of new hit-like matter, it has the potential to improve dataset quality by correcting labels, hence reducing misclassification noise. This increase in performance of an RF model before and after the updating of labels of the OriginalRefined-957 set from the Retrospective-97 results is seen in Fig. S10 where the PR AUC values of the RF model for any data subset increased significantly. In this context, denoising and exclusion of clear outliers are best viewed as steps that may improve xSAR model calibration and interpretability, providing greater confidence in feature–outcome associations and potentially strengthening guidance for compound prioritisation. However, such steps require an additional round of experiments, which ultimately slows the Design–Make–Test cycle, whose aim in early hit-expansion phases is often to improve potency rapidly and efficiently.^71^

While this method is tractable and conceptually intuitive (Fig. 3), it likely simplifies complex causal networks between chemical features and binding pose outcome. One interesting finding was that methylpiperazine containing compounds were, initially, associated with the diving pose and yet resolved later in the lateral pose (Fig. 5 and S9). Similarly, virtual screening compounds were resolved in new unpredicted poses (Fig. 8). As larger and more complex crystallographic datasets emerge, thanks to technological advancements,^31,72^ the application of more sophisticated analytical methods will be useful to extract more complex xSAR models. Indeed, such analytics, which are often machine learning-based, have already been developed and applied to DEL screenings of billions of compounds.^19^

One difficulty, here, is associating relevant chemical features to binding outcomes and poses from an incomplete combinatorial experiment of R groups. Not all R group combinations were initially enumerated, successfully synthesised and put forward in the crystallographic assay, as expected with any HT experiment. This challenge is exemplified by the unequal fraction of the methylpiperazine group present in the original robotic synthesis chemical space compared to the piperazine group (Fig. 1d). Indeed, there were 282 compounds with a methylpiperazine, 510 with a simple piperazine and 165 with neither. Since the methylpiperazines were present at a lower rate and introduced a new binding pose (diving), there are fewer measurements to enable a confident association between the chemical feature, methylpiperazine, and binding outcome compared to the lateral pose (Fig. 5). This is highlighted by the lower PR-AUC values for both diving PBS and NBS in the retrospective experiment (Fig. S11) and likely explains the lack of crystallographic diving binders identified in the prospective virtual screening exercise (Fig. 7). This limitation in combinatorial exploration also creates uncertainty, here, when trying to link chemical features to binding outcomes. The dataset and current analytics capture sets of features that consistently appear together or are absent together. This makes it difficult to isolate the impact of any single feature. It's important to remember that just because features are correlated does not mean they cause the same binding outcome. In fact, one feature in the correlated set may be a more prominent driver of a binding or non-binding event and corresponding pose, such as the methyl group off the piperazine potentially driving the adoption of the diving pose. Hence, this study also highlights the importance of systematic compound design to thoroughly and homogeneously cover chemical spaces from which analytics can build robust SAR models, and indeed there are algorithmic tools to support this systematic design for HT synthesis.^73,74^

In the prospective virtual screening experiment, there were differing hit rates for each pose scored sets between X-ray crystallography and the kinetic assay (Fig. 8). In the crystallographic experiments, there were more binders resolved from the lateral pose scoring whereas stronger kinetic binders were selected by the diving pose scores. This difference could be due to varying conformations of protein in solution *versus* crystal.^75^ Compounds with features associated with the diving pose could have higher affinity with conformations in solution whereas the crystal system may be more tolerant to the lateral pose. Additionally, our xSAR model, built solely on crystallographic data, inherently predicts crystallisation success rather than solution-phase binding, with stronger predictive power for the Prospective-93 set for lateral poses possibly due to their higher representation in the OriginalRefined-957 set (19 *vs.* 3 binders).

Performing co-crystallisation experiments could result in a higher rate of agreement between kinetic and structural hits,^75^ but was beyond the aim of this study. Here, we refer specifically to co-crystallisation using pure compounds, as the prospective experiment employed purchased, purified compounds rather than CRMs. Although co-crystallisation with CRMs could in principle be attempted, their inconsistent composition makes them unsuitable for reproducible crystallisation in the same space groups required for PanDDa analysis.^68^ These findings highlight the potential discrepancies between crystallographic and kinetic assay SAR, a known area of divergence.^76^ Having both kinetic and structural readouts on the same CRMs would enable a more direct comparison between binding affinity and pose. While our proof-of-concept kept these modalities separate, integrating them in future studies could further enhance the robustness of xSAR analyses and improve compound prioritisation.

This xSAR methodology can be applied to any other protein crystal system with examples of binder and non-binder ligand data, although an understanding of the limits of the ligands and system must be considered. Some scaffold conservation is required which is this method is particularly suited to fragment expansions. Hence, xSAR modelling may also be suited for fragment linking or merging strategies but were not addressed here. If applying to a project with less systematic ligand R-group exploration, there would be larger chemical differences between binders and non-binders, and therefore it would be more difficult to draw confident conclusions about the exact chemical modifications that resulted in the opposite crystallographic outcome. The R-group exploration could be augmented synthetically with a compound set with the R-groups explored, and then binders identified with established template-based docking procedures using resolved holo-conformation structures to update the CBB and CNB sets.^77^ Additionally, if applying to a protein crystal system with greater conformational flexibility, scores could be grouped for each conformational state (such as the grouping of diving and lateral poses in this work which saw a conformational change in the protein), then a consensus scoring scheme would be used to output the final score value.

## Conclusion and future directions

Here, we present a proof-of-concept for extracting crystallographic structure–activity relationships (xSAR), a systematic approach to analyse both positive and negative binding outcomes from HT crystallographic evaluation of CRMs. This method demonstrates the direct utilisation of comprehensive crystallographic data by calculating conservation scores of chemical features to distinguish binders from non-binders across different poses. Within purification-agnostic and other direct-to-biology workflows, our xSAR framework illustrates how HT crystallography datasets can be exploited more fully by integrating both binding and non-binding CRM outcomes into a crystallographic SAR model. We demonstrated this proof-of-concept by retrospectively identifying 26 false negatives and prospectively discovering novel crystallographic and kinetic binders through virtual screening, showcasing how large-scaled structural readouts can be directly leveraged without purification steps.

Looking forward, the xSAR model could be enhanced by integrating different bit weighting schemes such as down-weighting frequently occurring bits (*e.g.*, bits corresponding to aliphatic carbons) (eqn (S1) and (S2)) or assigning weights based on predicted relative binding free energy values (eqn (S8)). Utilising more advanced analytics, such as consensus scoring or machine learning approaches, to better capture the complex relationships between chemical features and binding outcomes could also be explored. This improved workflow could help prioritise compounds for robotic synthesis, with subsequent CRMs evaluated through both X-ray crystallography and GCI. Similarly, the combination of high-quality crystallographic information and simulated dynamic data could allow for off-rate predictions,^78^ providing orthogonal validation to off-rate crudes evaluation by GCI, hence also providing an opportunity for rationalising and denoising solution assays. The joint validation of CRMs *via* X-ray crystallography and GCI would provide complementary and mutually validating datasets to better guide design decisions. The current computational approach presented here establishes a baseline for subsequent method development, with additional formalisms for bit and score weighting available for future implementation seen in the SI.

Overall, this combination of using the xSAR from HT crystallography combined with kinetic methods, physics-based modelling and machine learning have the potential to further streamline Design–Make–Test cycles, accelerating the transition to the next design stage and confidently expediting early hit optimisation workflows.

## Methods

### Small molecule fingerprinting and dimensionality reduction

Feature-based invariant Morgan fingerprints were generated using a length of 2048 and a radius of six with RDKit (v2022.03.3).^79^ Fingerprints were used as input for bit scoring and feature extraction for t-Distributed Stochastic Neighbourhood Embedding using scikit-learn.^80^ The fingerprint bit vectors were embedded in two dimensions allowing for visualisation using the Jaccard distance metric with a perplexity of 30. Chemical similarity calculations of molecule pairs were performed using RDKit (v2022.03.3).^79^

### Bit and compound scoring methodology

The ligand dataset (D) was divided into two disjoint subsets: binders (B) and non-binders (NB), where:D = B ∪ NB, B ∩ NB = *Ø*

Each compound (*x*_k_), where *x*_k_ ∈ D was encoded as a binary fingerprint *x*_*k*_ = (*b*_1_, *b*_2_, …, *b*_*M*_) of length *M*, with *b*_m_ ∈ {0, 1}, indicating the activation status of bit, *b*, at index, *m*.

The Binding Bit Conservation Score (Sm^B^) for a bit *b*_m_, is calculated based across binder (B):1
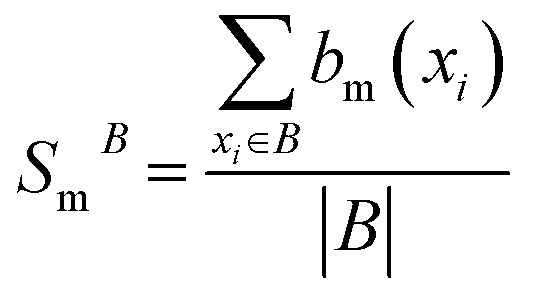
where |*B*| is the total number of binders, and 
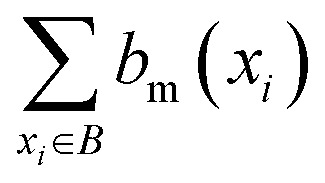
 represents the number of binders in which the bit, *b*_m_, is active. The Bit Conservation Scores (*S*_m_), range between 0 and 1, where *S*_m_ = 1 denotes perfect conservation of activation across binders, and *S*_m_ = 0, denotes a complete lack of bit activation across binders, for a given bit, *b*_m_.

Each bit, *b*_m_, was assigned to one of four mutually exclusive categories based on its corresponding Bit Conservation Score and activation status in non-binders (NB):

Conserved Binding Bits (CBB) are defined as:*b*_m_ ∈ CNB ⇔ Sm^B^ = 1meaning that the bit, *b*_m_, is fully conserved (always active) across binders (B) independently of its activation status in non-binders.

Conserved Non-binding Bits (CNB) are defined as:*b*_m_ ∈ CNB ⇔ Sm^B^ = 0 and ∃*x*_*j*_ ∈ NB, *b*_m_(*x*_*j*_) =1meaning that bit, *b*_m_, is never active in binders Sm^B^ = 0 but is active in at least one non-binder compound (∃*x*_*j*_).

Unconserved Bits (UCB) are defined as:*b*_m_ ∈ UCB ⇔ 0 <Sm^B^ < 1meaning that bit, *b*_m_, is partially conserved, meaning it is active in some binders but not all 0 <Sm^B^ < 1.

Unsampled Bits (USB) are defined as:*b*_m_ ∈ USB ⇔ 0 <Sm^B^ < 0 and ∀*x*_k_ ∈ D, *b*_m_(*x*_*j*_) = 0meaning that bit, *b*_m_, is never active in both binders and non-binders, implying a total deactivation across all compounds (*b*_m_(*x*_*j*_) = 0 ∀*x*_k_ ∈ D).

Therefore, the sum of all categorised bits equals the fingerprint length:|CBB| + |CNB| + |UCB| + |USB| = MThen, the Conserved Binding Bits set |CBB| was used to define the Positive Binding Score for a compound *x*_k_(PBS(*x*_k_)):2
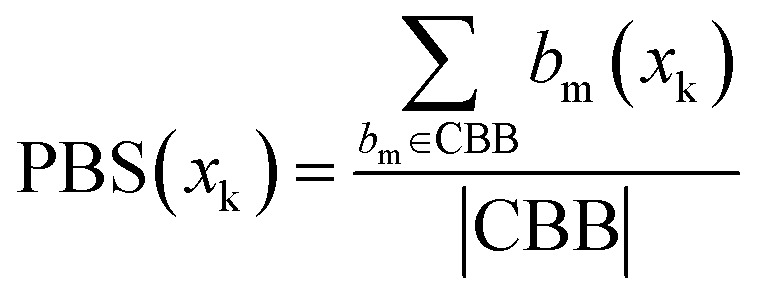
where 
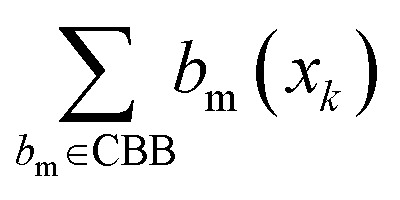
 represents the number of activated bits within the conserved binding bit set for compound *x*_k_, and |CBB| is the total number of bits in the conserved binding bit set. This score measures the degree to which a compound activates conserved binding bits.

Similarly, the Conserved Non-binding Bits set |CNB| was used to define the Negative Binding Score for a compound *x*_*k*_ (NBS(*x*_*k*_)):3
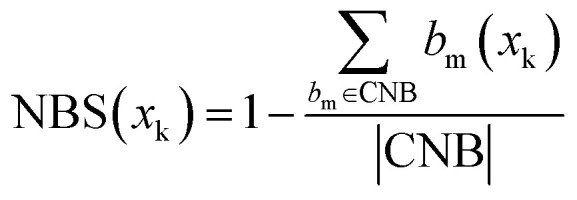
where 
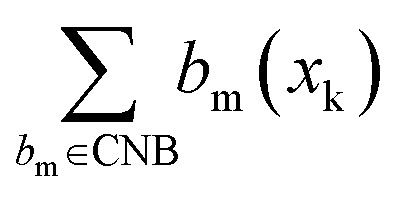
 represents the number of activated bits within the conserved non-binding bit set for compound *x*_k_, and |CNB| is the total number of bits in the conserved non-binding bit set. This score measures the degree to which a compound lacks activated conserved non-binding bits.

Finally non-binding or external compounds were classified based on their positive (PBS) and negative (NBS) binding scores:

• PBS(*x*_*k*_) = 1: compound *x*_*k*_ is a predicted a binder (*i.e.* a binder).

• PBS(*x*_*k*_) < 1: compound x_*k*_ is not a predicted binder (*i.e.* a non-binder).

• NBS(*x*_*k*_) = 1: compound *x*_*k*_ is not a predicted non-binder (*i.e.* a binder).

• NBS(*x*_*k*_) < 1: compound *x*_*k*_ is a predicted non-binder (*i.e.* a non-binder).

### Retrospective set selection

A subset of the OriginalRefined-957 dataset was selected by the following scoring process leading to the Retrospective-97 dataset. This process was applied to the three pose datasets (All, Diving, Lateral) for each score (PBS and NBS), separately. (1) Filter out compounds that are not available in Enamine (Fig. S5); (2) select compounds with score equal to 1 to buy; (3) rank remaining compounds (with scores less than 1); (4) split the ranked compound gradients into 25 bins, with log ordering of increased sampling towards higher scores; (5) for each bin, select the cheapest compound, and if prices are the same, choose the one with the score closest to the bin's average.

This process resulted in selecting 150 total compounds (25 compounds per ensemble, *i.e.* all PBS, all NBS, diving PBS, diving NBS, lateral PBS, lateral NBS), with 97 compounds being purchased as there was overlap between ensemble selections, referred to as the Retrospective-97 set. Hence, the compounds in the Retrospective-97 dataset were scored using the 6 possible scoring schemes (3 pose-based binding bits by 2 scores) resulting in all compounds being used to evaluate the scores regardless of which ensemble they were selected from (Fig. 4).

### Virtual screening

The following virtual screening procedure was performed twice, once using the lateral pose scoring metrics and once using the diving pose scoring metrics (Fig. 7). The filtering criteria were chosen to produce a manageable set for analysis.

The screening was performed using a subset of the Enamine REAL© database^51,52^ downloaded in early 2021, totalling approximately 1.7 billion compounds. First, the catalogue was filtered to retrieve the top 45 000 compounds based on PBS, excluding those which violated the Rule of Five to select lead-like compounds.^53,54^ Then compounds with a NBS greater than or equal to the average were removed to exclude compounds containing non-binding features. The tool, CoPriNet^81^ was used for price prediction and the 15 000 cheapest scoring compounds were selected for further processing. A diversity selection was performed to select the 10 000 most diverse compounds, based on the Tanimoto distance, using the lazy MaxMin diversity picker implemented in RDKit. This process resulted in the selection of 10 000 compounds per pose.

Binding pose predictions were then performed to further filter this set using the previously resolved high-resolution protein crystal structures bound with reaction products from CRMs.^30^ All water molecules were removed and crystalised reaction product ligands were extracted with MDAnalysis.^82,83^ Missing protein heavy atoms and hydrogens were added with PDB2PQR^84,85^ using PARSE as force field,^86^ PROPKA^87,88^ for protonation an assuming physiological pH of 7.4. Crystalised template ligands were protonated with Quacpac Toolkit (OpenEye).^89^ These prepared crystallised ligand and protein templates are used in the following steps.

Compound poses were calculated using the tool Fragmenstein^58^ with default parameters. For each compound, a crystallised ligand structure and protein template chosen to place each compound was selected by the highest Tanimoto similarity to a crystallised ligand. Then, compounds were filtered based on values of the ligand energy ratio to the crystallised ligand structure as described in Wills *et al.*^90^ From this filtered set, ΔΔ*G* values were calculated using the protein template and compounds in the top 3% (lowest ΔΔ*G* values) were selected.

Finally, the same diversity selection applied previously was performed, resulting in 47 compounds chosen scored by the lateral pose and 46 chosen and scored by the diving pose.

### Protein expression, purification and crystallisation

BL21 cells containing a pNIC28-Bsa4 vector coding for PHIP2 (UniProt ID: Q8WWQ0) were taken from a glycerol stock. 2 mL of Luria Broth pre-culture with 50 µM kanamycin were inoculated into 1 L Terrific Broth media with 2% glycerol (v/v), 0.01% (v/v) of 10% (v/v) sigma Antifoam 204 in ethanol, 50 µM FeCl_3_, 20 µM CaCl_2_, 10 µM MnCl_2_, 10 µM ZnSO_4_ and 2 µM of CoCl_2_, CuCl_2_, NiCl_2_, Na_2_MoO_4_, Na_2_SeO_3_ and H_3_BO_3_, 2 mM CaCl_2_, 25 mM(NH_4_)_2_SO_4_, 2.77 mM glucose and 50 µM kanamycin. The cultures were grown for 6 h at 37 °C at 250 rpm. PHIP(2) expression was induced overnight at 18 °C with 0.1 mM IPTG. Cultures were centrifuged at 4000 g for 30 minutes at 4 °C.

Pellets were resuspended in lysis buffer (10 mM HEPES, 500 mM NaCl, 5% glycerol, 0.5 mM TCEP, 0.5 mg mL^−1^ Lysozyme, 1 µg mL^−1^ Benzonase, pH 7.5). The solution was vortexed and left at room temperature for 30 min before. 2% (v/v) triton-X- and 20 mM imidazole finale concentrations were added to the mixture before being centrifuged at 4000 g for 30 min at 4 °C. The supernatant was applied onto a 1 mL His GraviTrap columns (GE healthcare) fitted with a LabMate extender. The columns were washed twice with wash buffer (10 mM HEPES, 500 mM NaCl, 5% Glycerol, 0.5 mM TCEP, 20 mM imidazole, pH 7.5). The columns were slotted PD10 columns fitted with LabMate extenders. The proteins were eluted by applying 2.5 mL of elution buffer (10 mM HEPES, 500 mM NaCl, 5% glycerol (v/v), 0.5 mM TCEP, 500 mM Imidazole, pH 7.5) onto each GraviTrap column. 3.5 mL of wash buffer was applied onto each PD10 column and elutions were collected. 1 OD_280_ unit of TEV protease per PHIP(2) 10 OD_280_ units was added to the elutions and incubated at 4 °C. The solutions were run back over His GraviTrap columns as mentioned above. The fractions were concentrated by 20-fold and applied onto a Yarra SEC 2000 pre-equilibrated with wash buffer. The fractions containing the protein were collected using either a biorad C-9 or a Cytiva ALIAS. The fractions were concentrated to about 15 mg mL^−1^ of protein and flash-frozen in liquid nitrogen.

PHIP(2) was crystallised in space group C2 at 4 °C by vapour diffusion in 230 nL sitting drops, by mixing 100 nL protein in wash buffer with 100 nL reservoir buffer (20% PEG8000 and 40 mM potassium phosphate) and 30 nL seeds of the same composition than reservoir with final pH measured to be about 5.6.

### Crystallographic evaluation

All compounds used for the retrospective (Retrospective-97) and prospective (Prospective-93) evaluation were purchased from Enamine and stored in ethylene glycol at a concentration of 40 mM.

Small molecule crystallographic screening was performed at XChem in Diamond Light Source (Harwell, UK).^31,32^ Crystals suitable for soaking were located in the plates with TexRank.^91^ Suitable crystals were targeted with compounds with acoustic liquid dispensing using an ECHO.^92^ The crystals were incubated for 24 hours, at 4 °C, at a final compound concentration of 8 mM resulting in a 20% (v/v) ethylene glycol final concentration in the drops. The XChemXplorer^93^ was used for crystallographic workflow management and paralleling. Molecular replacements and initial refinements were performed with DIMPLE. PanDDA^68^ was used to identify low occupancy binding events. Ligands were fit in Coot and the structures refined with Buster^94^ and/or Refmac^95^ before deposition on the Protein Data Bank (PDB). Structural models and crystallographic statistics can be retrieved from the PDB deposition ID: G_1002265.^96^

### Grating-coupled interferometry assay

Pulsed single-concentration surface-based biophysical measurements of binding kinetics were performed using a Creoptix® WAVE system (Creoptix®, AG).^56^ A sensor chip was conditioned using injections of borate buffer (10 mM sodium tetraborate pH 9, 1 M NaCl). The sensor chip was activated using 1 : 1 mixture of 400 mM EDC/100 mM NHS for 420 s at 10 µL min^−1^. PHIP(2) was diluted in sodium acetate buffer (10 mM, pH 5.0) in to 5 µg mL^−1^ and injected over the active surface at a flow rate of 10 µL min^−1^ for immobilisation to a final level of 6554 surface mass (pg mm^−2^) corresponding to an injection time of 420 s. The surface was then deactivated with ethanolamine-HCl (1.0 M pH 8.5) for 420 s.

Kinetic analysis for PHIP(2) and small molecules was performed using a pulsed injection scheme (waveRAPID®)^56^ at 25 °C with a 5 s association and 20 s dissociation at a top concentration of 200 µM for all compounds. Blank samples of the running buffer were injected during the measurements every fifth cycle. The running buffer was composed of 20 mM HEPES, pH 7.5, 50 mM NaCl, 0.5% (v/v) Tween-20, and 0.5% (v/v) ethylene glycol. Compounds were applied to the immobilised surface and a reference channel. Data analysis and visualisation were performed using the WAVEcontrol® software 4.5.13 (correction applied: X and Y offset; DMSO calibration; and double referencing). Kinetic parameters were calculated using the Direct Kinetics fitting engine with 1 : 1 kinetic binding model. This fitting approach can lead to suboptimal fits in some cases, but more complex fitting or parameter optimisation was deliberately avoided to enable rapid evaluation.

The WaveRapid® GCI analysis method quantifies the error associated with both *k*_a_ and *k*_d_ values, expressing them as a percentage relative to the measured values. Consequently, these must be manually propagated to estimate *K*_D_ error value to properly estimate the affinity range of a particular compound for the target. The errors were propagated using root-sum-square method. The equilibrium constant error is calculated as:4
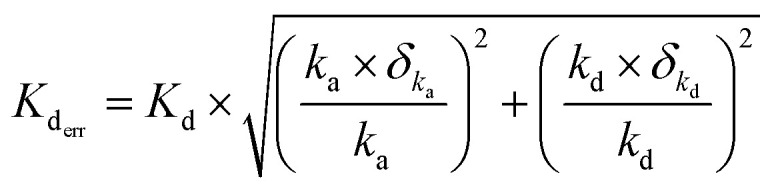
where *K*_d_ and *K*_d_err__ are the equilibrium dissociation constant and associated error, respectively. *k*_a_ and *δ*_*k*_a__ are the association rate and, associated error expressed in percent, respectively. *k*_d_ and *δ*_*k*_d__ are the dissociation rate and, associated error expressed in percent, respectively.

Binding events were further considered if both the *δ*_*k*_a__ and *δ*_*k*_d__ were lower than 25% and paired with a maximum observed binding signal (*R*_max_) value greater than 10. Although no universally accepted hit-calling criteria exist, the thresholds mentioned above are relatively stringent and applied to potentially minimise the likelihood of false positives.

Ligand efficiency (LE) was computed to assess the efficiency of molecular interactions relative to the number of heavy atoms in each compound. LE values were calculated according as follows:5
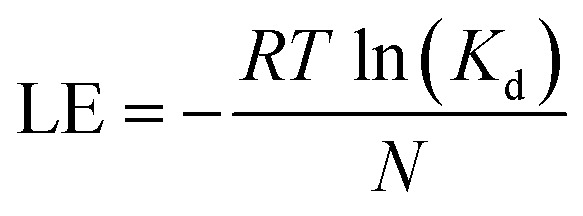
where *R* is the gas constant and equals to 1.987 × 10^−3^ kcal/(mol K). *T* is the temperature and equals 298 kelvin. *K*_d_ is the equilibrium dissociation constant, measured *via* GCI, and expressed in molar (*M*). *N* is the number of heavy atoms as was determined from the SMILES representation of each molecule using RDKit. The final ligand efficiency (LE) has units of kcal/(mol × heavy atom).

## Author contributions

Harold Grosjean and Kate K. Fieseler wrote the manuscript and developed the cheminformatic methodologies. Harold Grosjean and Rubén Sanchez-Garcia designed and executed the virtual screening studies. Harold Grosjean performed protein expression and purification, X-ray crystallography, structure refinement and kinetic binding analysis. Frank von Delft, Philip C. Biggin, Charlotte M. Deane, and Warren Thompson supervised the project and contributed to its conceptual and methodological design.

## Conflicts of interest

Charlotte M. Deane discloses membership of the Scientific Advisory Board of Fusion Antibodies plc and AI proteins, and is a founder of Dalton Tx. The remaining authors declared that this work was conducted in the absence of any commercial or financial relationships that could be construed as a potential conflict of interest.

## Supplementary Material

SC-017-D5SC04919A-s001

## Data Availability

The code and data are available in the following GitHub repository: https://github.com/haroldgrosjean/xSAR. The repository includes: • /xsar_model/data/1_OriginalRefined-957_BeforeReevaluation/: Initial binary binding outcomes for 957 compounds screened as crude reaction mixtures. • /xsar_model/data/2_Retrospective-97/: Corrected binding labels for 97 resynthesized and re-evaluated compounds. • /xsar_model/data/3_OriginalRefined-957_AfterReevaluation/: Full dataset of 957 compounds with updated labels for the Retrospective-97 subset. • /xsar_model/data/4_MethodsBenchmarking/: Datasets, model predictions, and hyperparameter optimization results used to benchmark ligand-based classifiers. • /xsar_model/data/5_virtual_screening/: Virtual screening data of the Enamine REAL database. • /xsar_model/data/6_experimental_validation/: GCI kinetic assay results for the 93 virtual screening compounds. • /xsar_model/scripts/: Python scripts to for computing the xSAR model. • /xsar_model/examples/: Sample workflows illustrating the scoring and classification pipelines. For all datasets, versions are provided for the full ensemble (All) as well as pose-specific subsets (Lateral and Diving). Detailed dataset descriptions and usage instructions are provided in the respective README.md files throughout the repository. Structural models and crystallographic statistics can be retrieved from the PDB deposition ID: G_1002265. Supplementary information (SI): additional analyses of feature/bit overlap and PBS/NBS score distributions, chemical-space visualisations, compound structures and outcomes for the Retrospective-97 rescreening and Prospective-93 virtual-screening follow-ups, GCI sensorgrams/kinetic parameters for hit validation, benchmarking against similarity and random-forest classifiers, and additional mathematical formalisms for score weighting and bit classification. DOI: https://doi.org/10.1039/d5sc04919a.
