# Supplementary material for: Structure–activity relationships can be directly extracted from high-throughput crystallographic evaluation of fragment elaborations in crude reaction mixtures
Source: Chem Sci. 2025 Dec 23;17(9):4571–93. doi: 10.1039/d5sc04919a (PMC12797250; doi:10.1039/d5sc04919a)
Supplement: SC-017-D5SC04919A-s001 [file SC-017-D5SC04919A-s001.pdf]

Supplementary Information (SI) for Chemical Science.
This journal is © The Royal Society of Chemistry 2025

Supplementary Information: Structure-activity relationships can be directly extracted from high-throughput crystallographic evaluation of fragment elaborations in crude reaction mixtures

Harold Grosjean[#1,2], Kate K. Fieseler[#3,4,5], Ruben Sanchez-Garcia[3,5], Warren Thompson[2,4], Charlotte M. Deane[3], Frank von Delft[2,4,5] and Philip C. Biggin[1]

# Joint first authors

1. Structural Bioinformatics and Computational Biochemistry, Department of Biochemistry, University of Oxford, South Parks Road, OX1 3QU, Oxford, United Kingdom.

2. Diamond Light Source Ltd, Harwell Science and Innovation Campus, OX11 0QX, Didcot, United Kingdom.

3. Oxford Protein Informatics Group, Department of Statistics, University of Oxford, Oxford OX1 3LB, United Kingdom.

4. Research Complex at Harwell, Harwell Science and Innovation Campus, OX11 0FA, Didcot, United Kingdom.

5. Centre for Medicines Discovery, University of Oxford, Oxford OX3 7DQ, United Kingdom.

Supplementary data

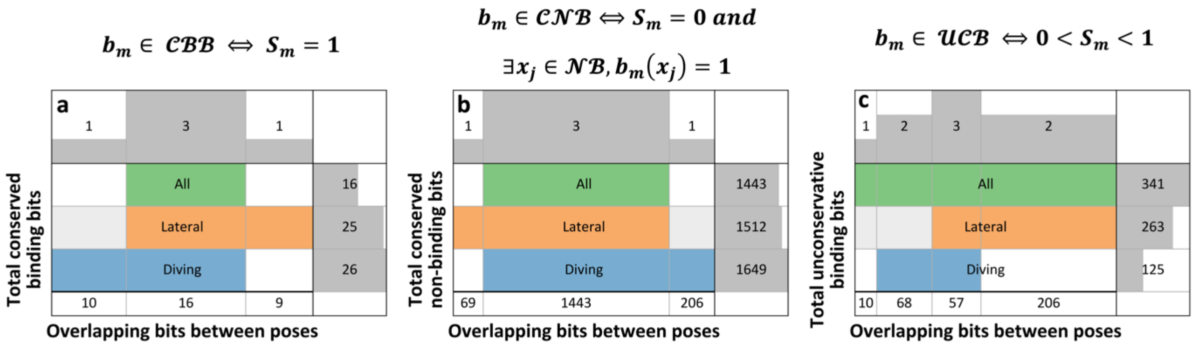

Supplementary figure 1 : Venn diagrams comparing poses within each bit classification label: (a) conserved binding bits ($\mathcal{CBB}$), (b) conserved non-binding bits ($\mathcal{CNB}$), and (c) unconservative bits ($\mathcal{UCB}$). Each Venn diagram is separated into five rows and four columns (five columns in (c)), showing the number of sub-ensembles the bits are present in, the total number of bits composing the intersection, and the bit ensemble for and intersections between the different poses. The first three columns relate to the different ensembles and subsequent overlaps, while the last column shows the total number of bits for a given pose. The condition defining the classification is showed above each Venn diagram. See Methods for exact definitions.

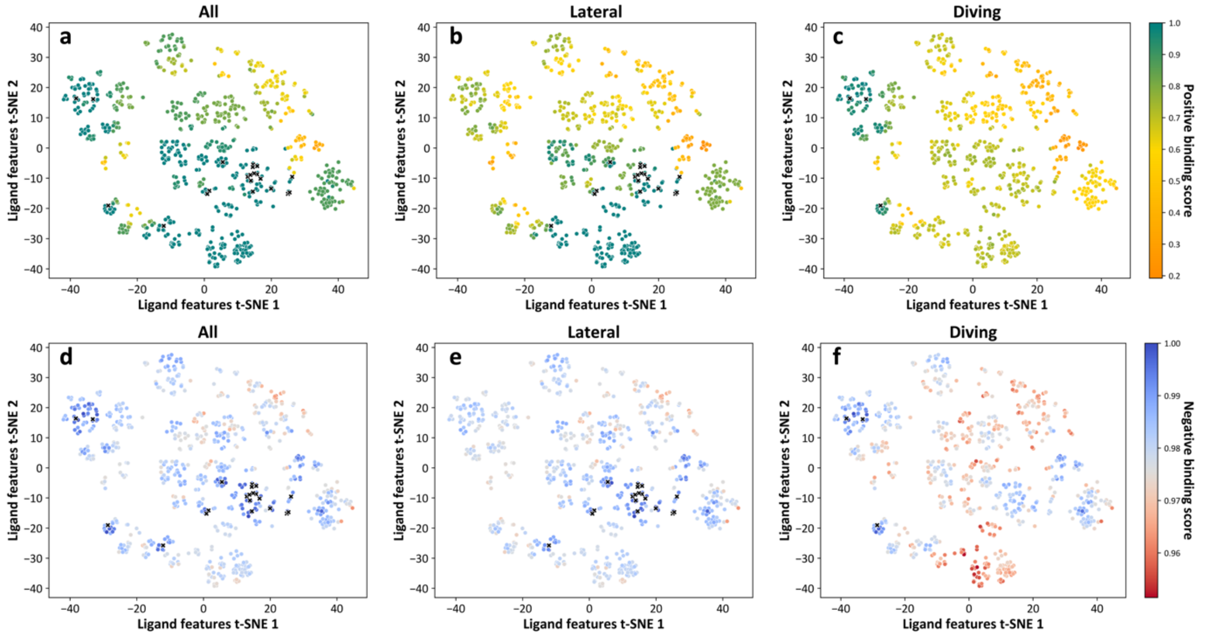

Supplementary figure 2 : Pose-specific Positive Binding Score (PBS) and Negative Binding Score (NBS) values of the OriginalRefined-957 compounds. The chemical space of the OriginalRefined-957 compounds projected to 2D using t-SNE dimensionality reduction of the fingerprints and colored by PBS (a–c) or NBS (d–f) values, labeled by pose dataset used ((a)(d) all, (b)(e) lateral, or (c)(f) diving). Resolved binders are shown with black crosses.

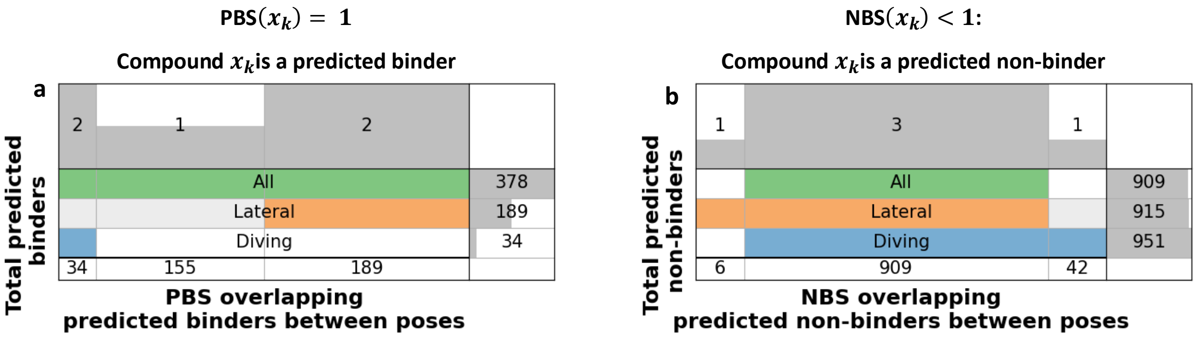

Supplementary figure 3: Venn diagrams comparing poses within each compound classification label: (a) likely and (b) unlikely binders. Each Venn diagram is separated into five rows and four columns, showing the number of sub-ensembles the compounds are present in, the total number of compounds composing the intersection, and the compounds ensemble for and intersections between the different poses. The first three columns relate to the different ensembles and subsequent overlaps, while the last column shows the total number of compounds for a given pose. The condition defining the classification is showed above each Venn diagram.

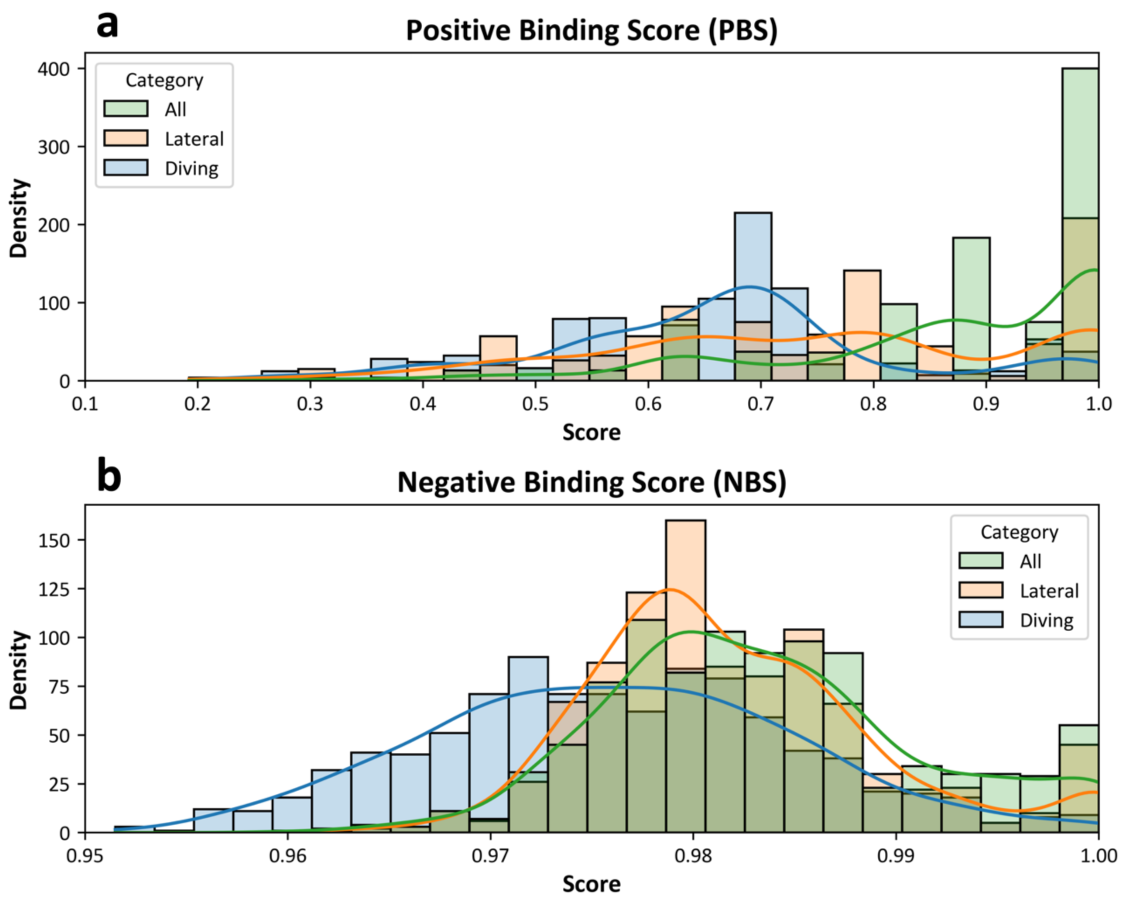

Supplementary figure 4: Distributions of the PBS and NBS values for the OriginalRefined-957 set. The top panel (a) shows the distribution of PBS values, which quantifies the presence of conserved binding bits, while the bottom panel (b) presents the NBS values which measures the absence of conserved non-binding bits. Each score is shown for the three pose sub datasets: all compounds (green), lateral binders (orange), and diving binders (blue). Kernel density estimates are overlaid to illustrate the smooth distribution of scores within each subset. The distinct distributions highlight how binding characteristics vary based on binding pose, with diving binders exhibiting a broader PBS distribution and lateral binders showing a more concentrated NBS distribution.

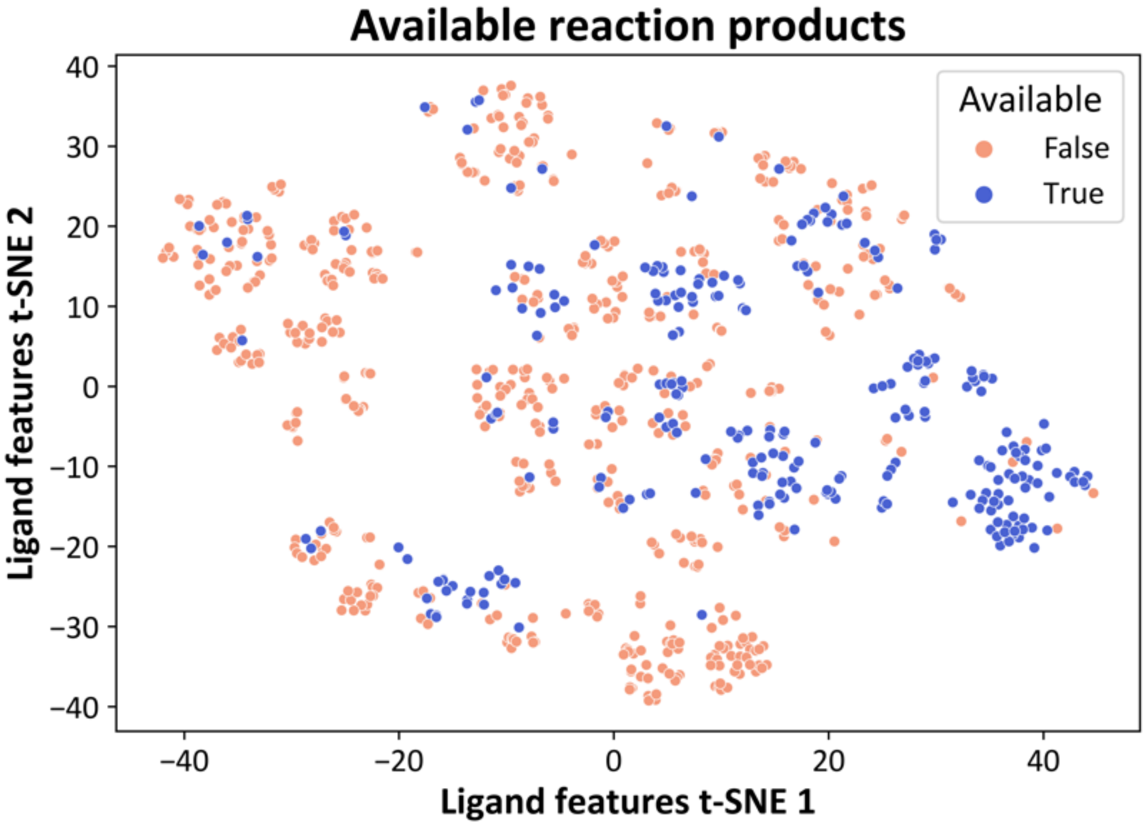

Supplementary figure 5: The compounds from the OriginalRefined-957 set that were initially enquired to Enamine for purchase in pure form for retrospective study. The available and unavailable compounds are showed in purple and pink, respectively.

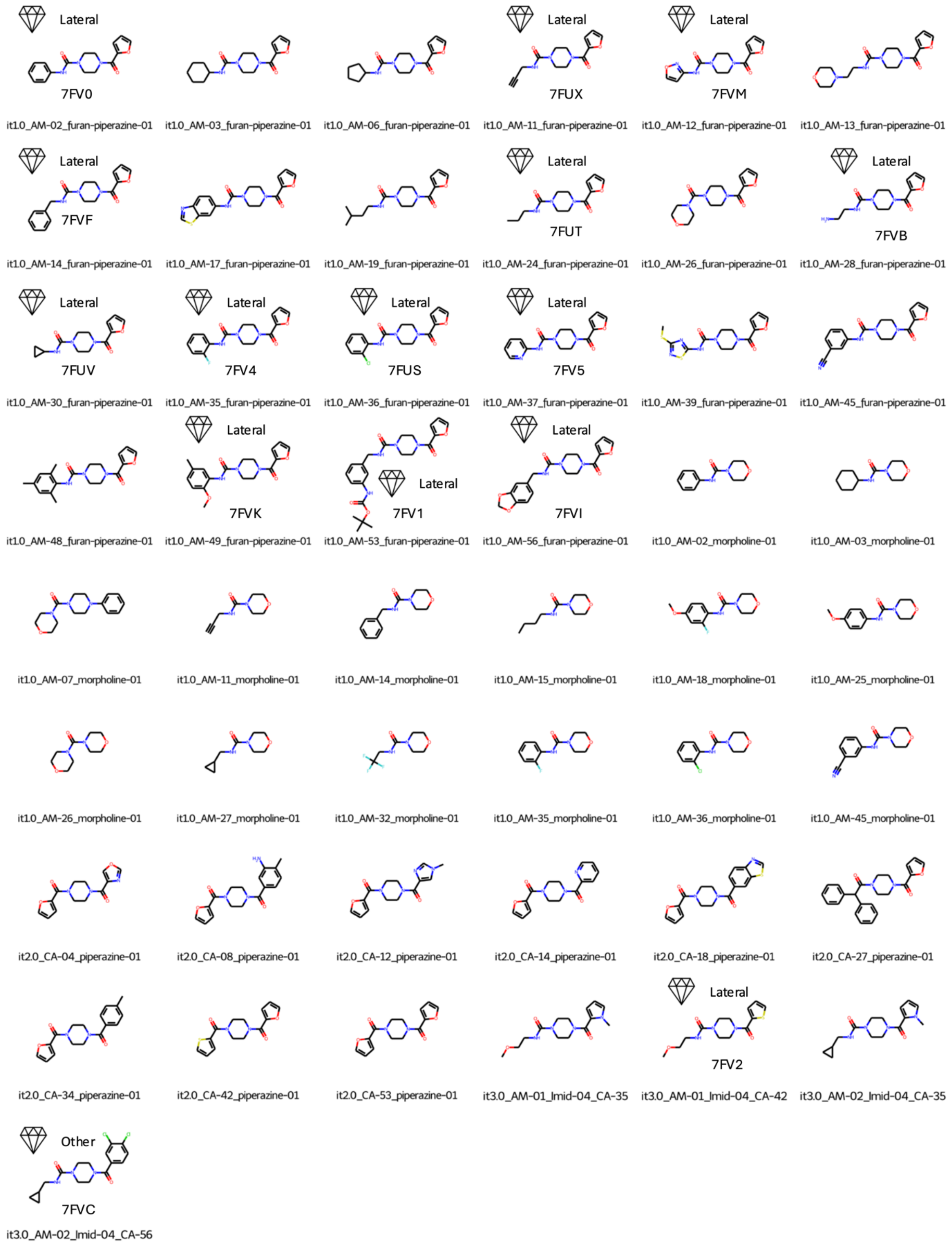

Supplementary figure 6.1: 2D structures of the first half of the Retrospective-97 set, robotic compounds purchased and rescreened in pure form for xSAR retrospective validation, benchmarking and dataset denoising. Displayed are the chemical structures of the Retrospective-97 set that were purchased in pure form and reevaluated by X-ray crystallography against PHIP(2). Each compound is labeled with its unique identifier and binding pose classification, with "Lateral" and "Diving" indicating compounds that adopted respective binding modes in the crystallographic assay. Diamonds denote crystallographically confirmed binders, while structures without diamonds represent non-binders in the rescreening. This dataset also serves as the test set for validation of the xSAR model, assessing its ability to recover false negatives and predict binding probability based on the PBS and NBS metrics.

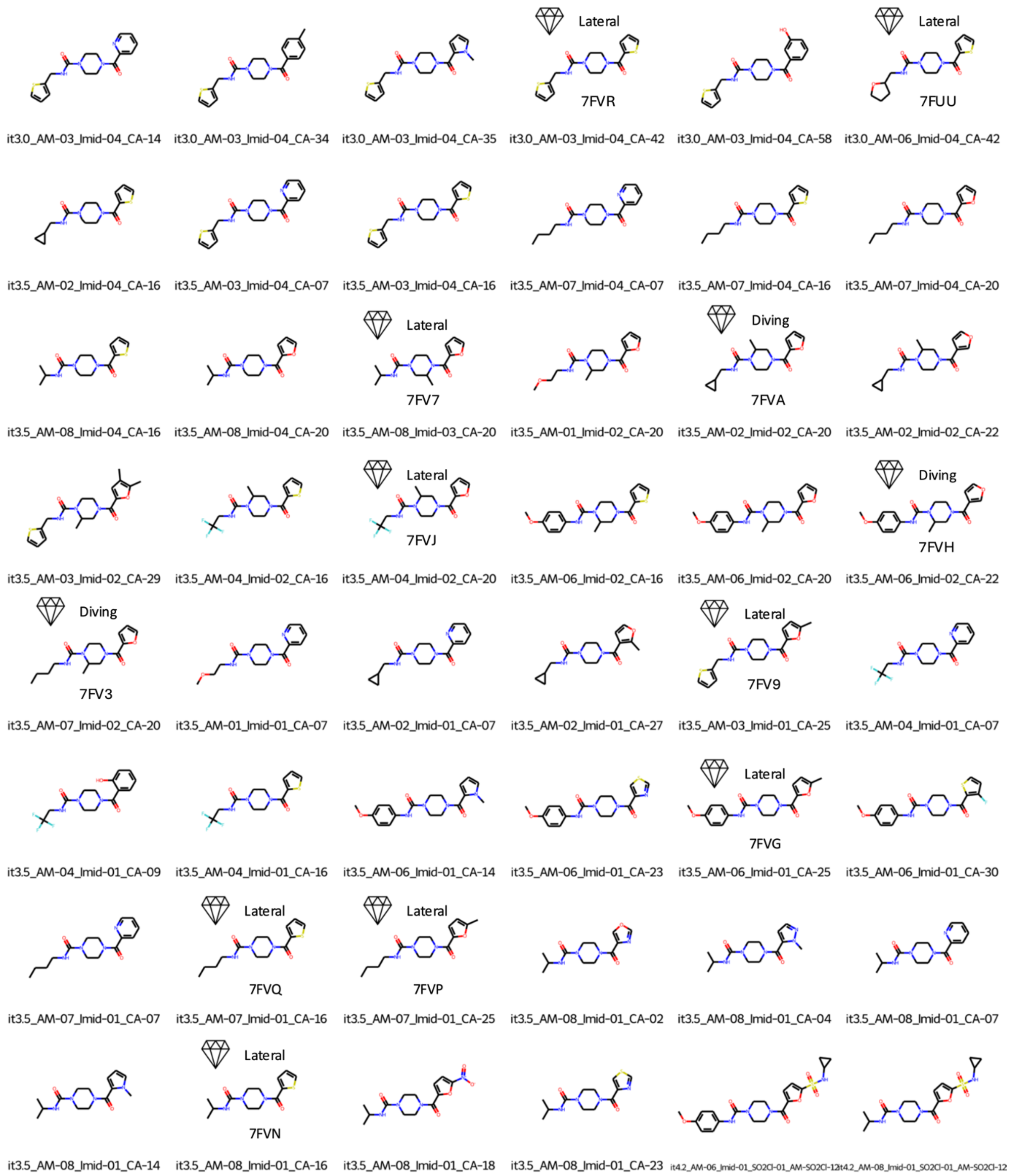

Supplementary figure 6.2: 2D structures of the second half of the Retrospective-97 set, robotic compounds purchased and rescreened in pure form for xSAR retrospective validation, benchmarking and dataset denoising. Displayed are the chemical structures of the Retrospective-97 set that were purchased in pure form and reevaluated by X-ray crystallography against PHIP(2). Each compound is labeled with its unique identifier and binding pose classification, with "Lateral" and "Diving" indicating compounds that adopted respective binding modes in the crystallographic assay. Diamonds denote crystallographically confirmed binders, while structures without diamonds represent non-binders in the rescreening. This dataset also serves as the test set for validation of the xSAR model, assessing its ability to recover false negatives and predict binding probability based on the PBS and NBS metrics.

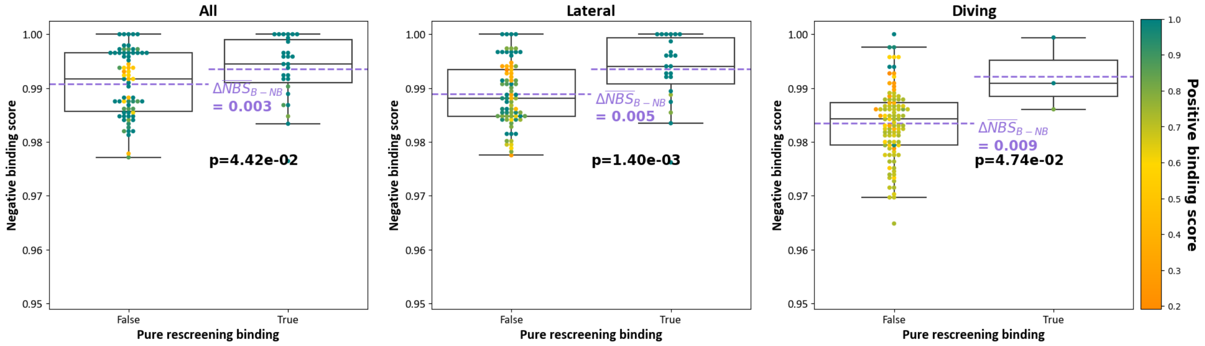

Supplementary figure 7: Rescreening analysis based on the NBS. This shows the enrichment analysis, with True and False categories representing the pure crystallographic rescreening binding outcome on the x-axis and the NBS value on the y-axis. Purple dotted lines show the average NBS for each class and the resulting difference (ΔNBS) between binders (B) and non-binders (NB). The p-values, calculated from a Mann-Whitney U test, indicate the significance of the metric in discriminating binders from non-binders. The points are coloured by PBS values from low (orange) to high (green).

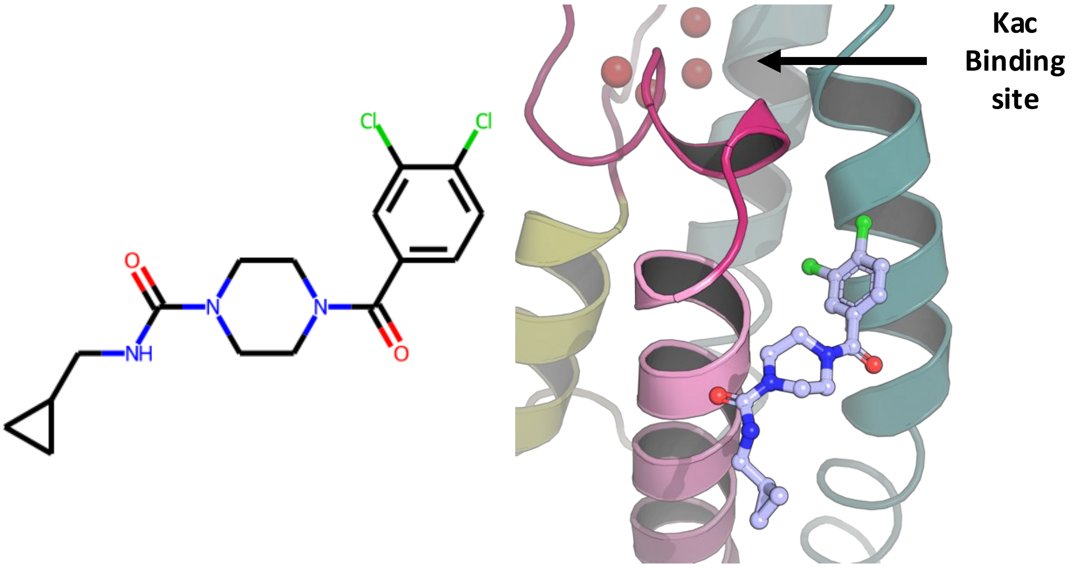

It3.0_AM-02_Imid-04_CA-56 7FVC

Supplementary figure 8: Binding compound (PDB ID: 7FVC) from screening the Retrospective-97 set not resolved within the pharmacologically relevant acetylated lysine binding site. The chemical structure of the compound is on the left and the pose of the compound in relation to the acetylated lysine binding site (Kac) is seen on the right.

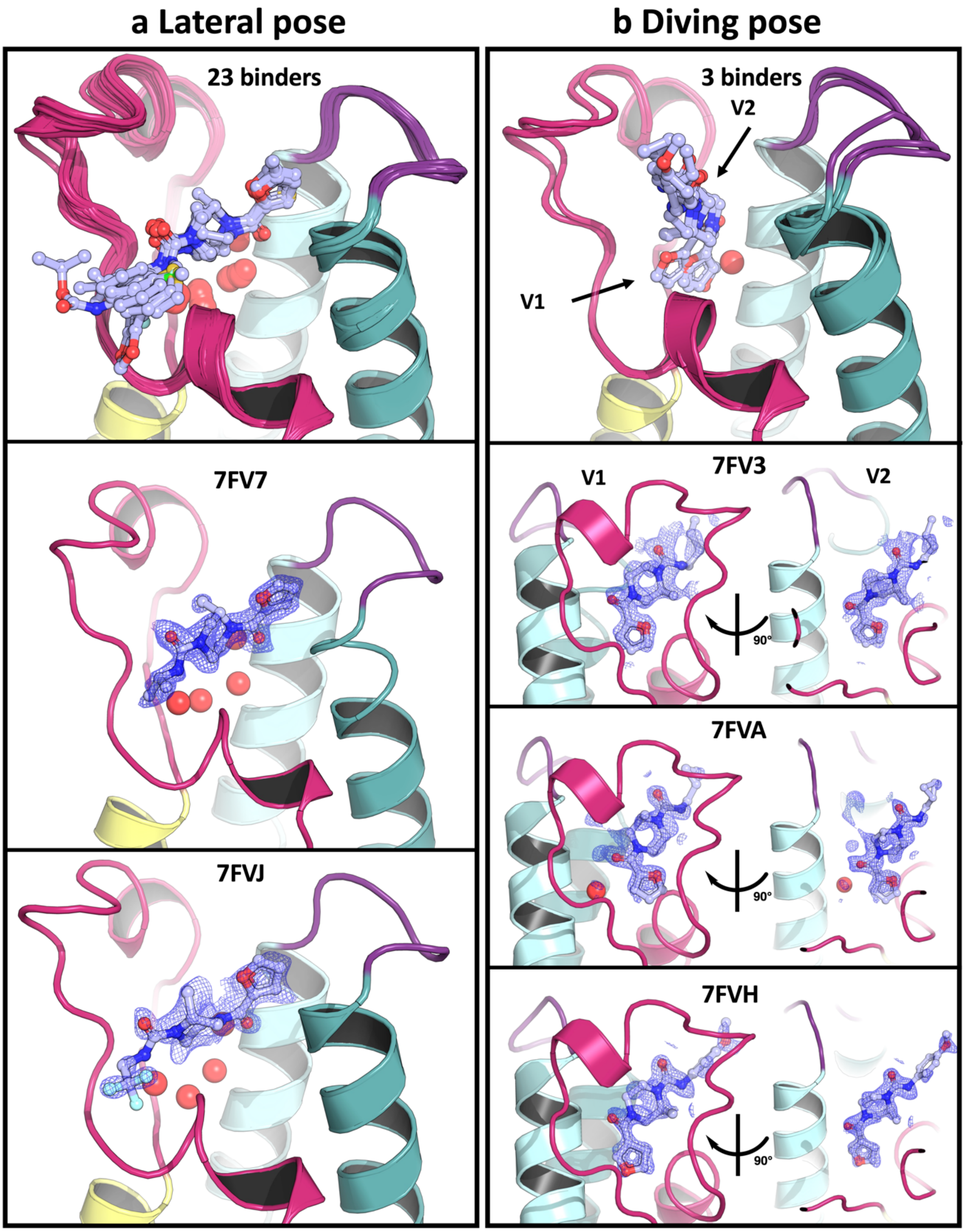

Supplementary figure 9: Rescreening of pure compounds (Retrospective-97) resolves false negatives and reveals outliers poses and chemotypes. The resolved binders in the (a) lateral pose and the (b) diving pose. The top panel of each column shows all resolved lateral and diving compounds (false negatives) from screening the Retrospective-93 set. The left column shows the structures and associated PanDDa event maps for lateral binding outliers bearing a methylpiperazine. The right column also shows structures and associated PanDDa event maps for all three diving binders.

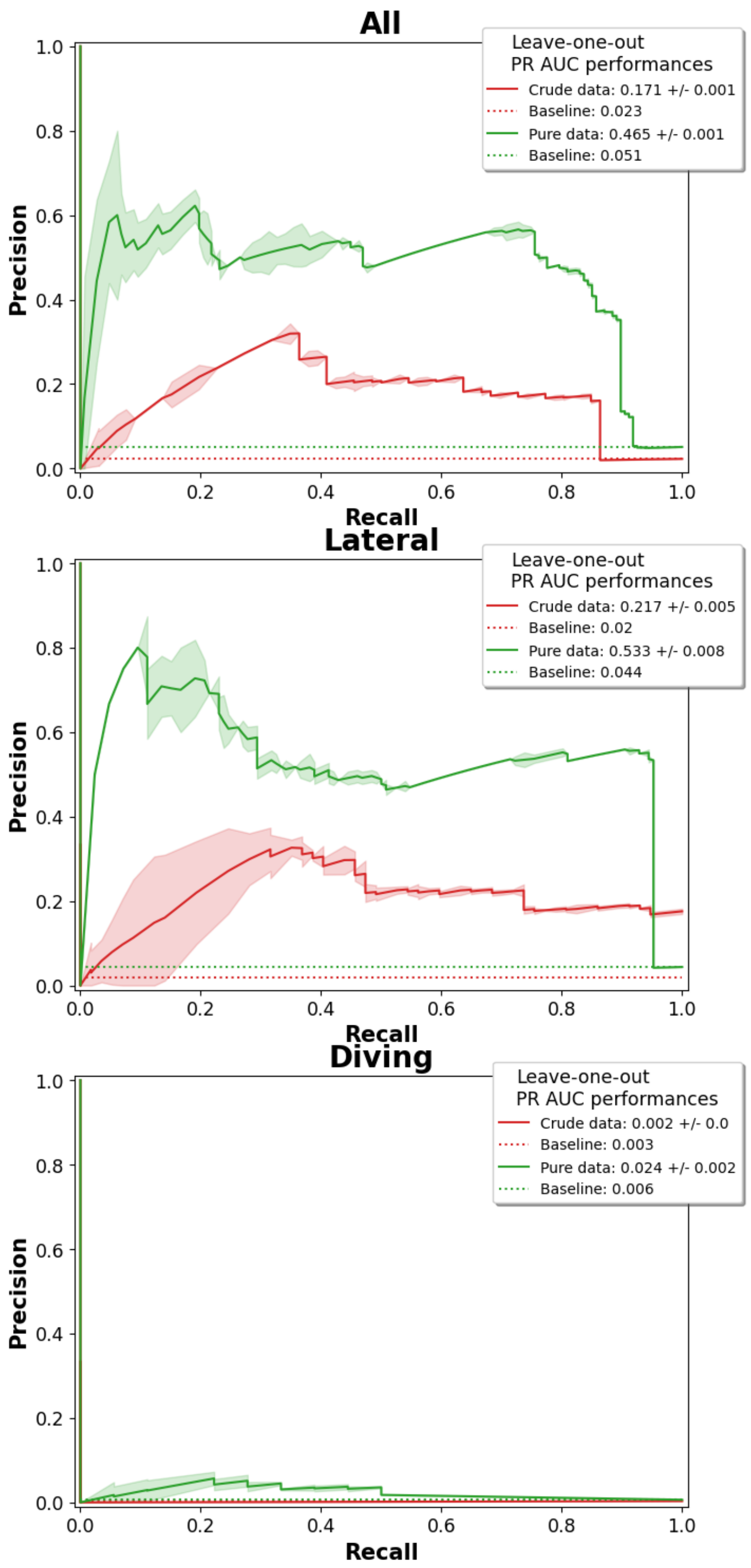

Supplementary figure 10: Leave-one-out validation of random forest classifier performance before and after rescreening experiment and correction of experimental false negative labels. A leave-one-out validation of the random forest classifier was applied to the OriginalRefined-957 dataset (red) and the same dataset where binding labels of the 26 false negatives are updated (green). The operation was done once for each dataset. The errors showed are related to the precision and the ones reported to the overall PR-AUC.

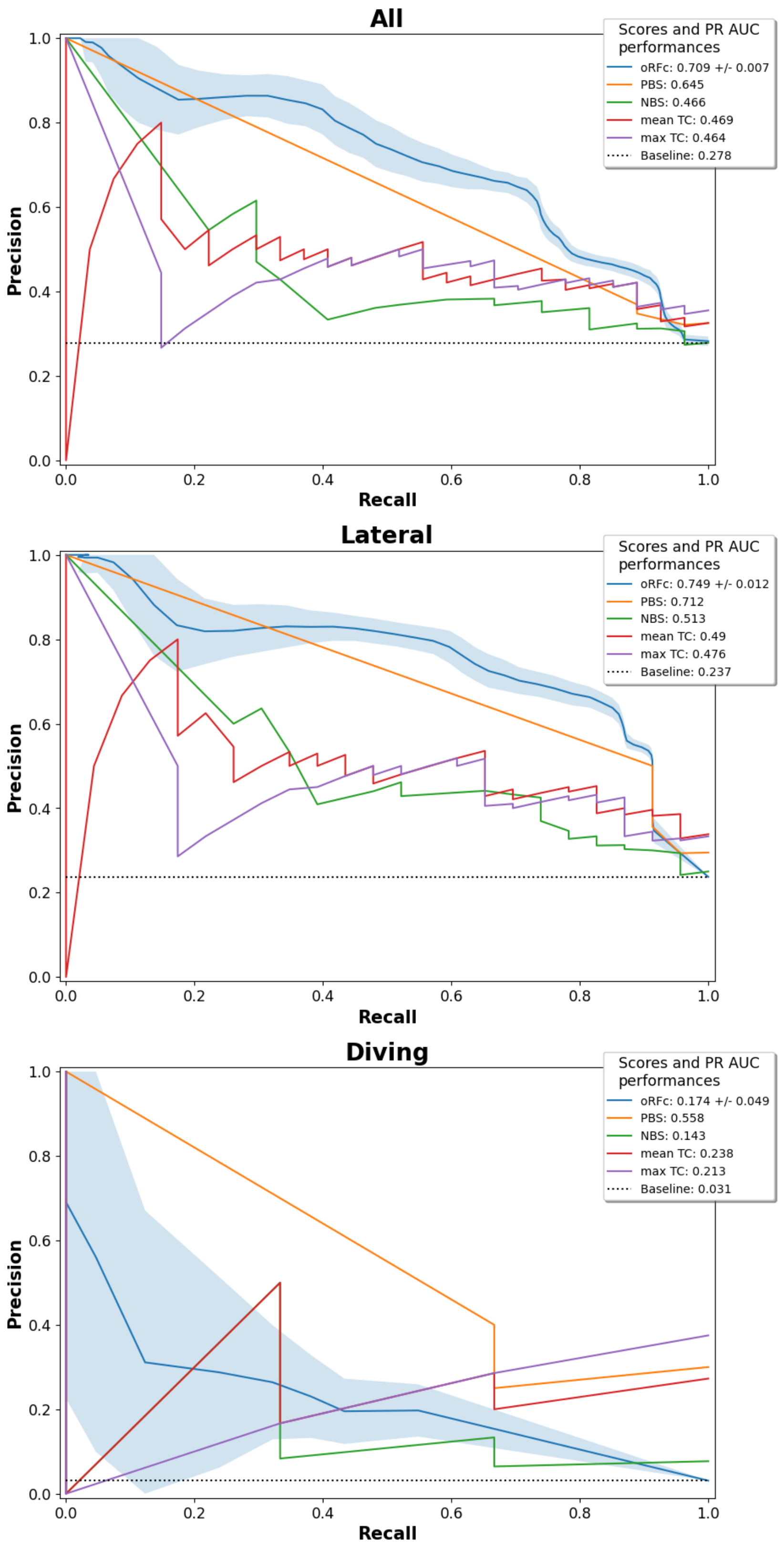

Supplementary figure 11: Precision-recall curves of methods applied to categorize binders and non-binders in the Retrospective-97 set. The methods of random forest (blue), PBS (orange), NBS (green), mean Tanimoto coefficient (TC) (red), and maximum TC (purple) applied to categorize binders in the Retrospective-97 set using the all dataset, lateral, and diving pose subsets. The random forest method resulted in the highest PR AUC values for the all set and lateral pose subset, whereas the PBS metric resulted in the highest PR AUC value for the diving pose subset. The performance of the random forest could be inflated as the hyperparameters were optimised using the Retrospective-97 set. The NBS and TC based methods had poor performance for all sets.

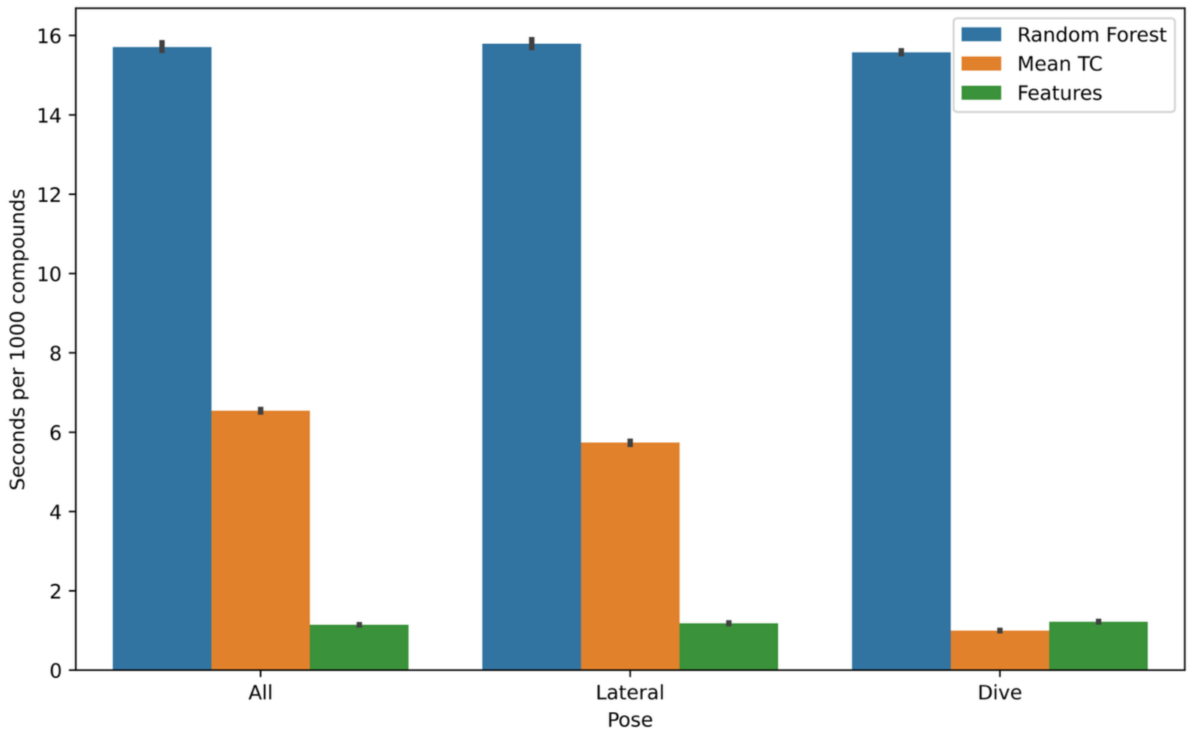

Supplementary figure 12: Feature-based scoring (PBS and NBS) is faster than random forest classification and is hit-rate independent. A speed benchmark was carried out to assess which method performs faster for ligand-based virtual screening. The random forest was slower than retrieving both PBS and NBS values for a given molecule (Features). The mean Tanimoto coefficient (TC) was slower than the feature method for the all and lateral data subsets as it is dependent on the number of positive. The mean TC was faster than the feature method for the dive subset as it only contains three binders. All computations were performed on a MacBook Pro using a single Apple M1 Pro chip and with 32GB of RAM.

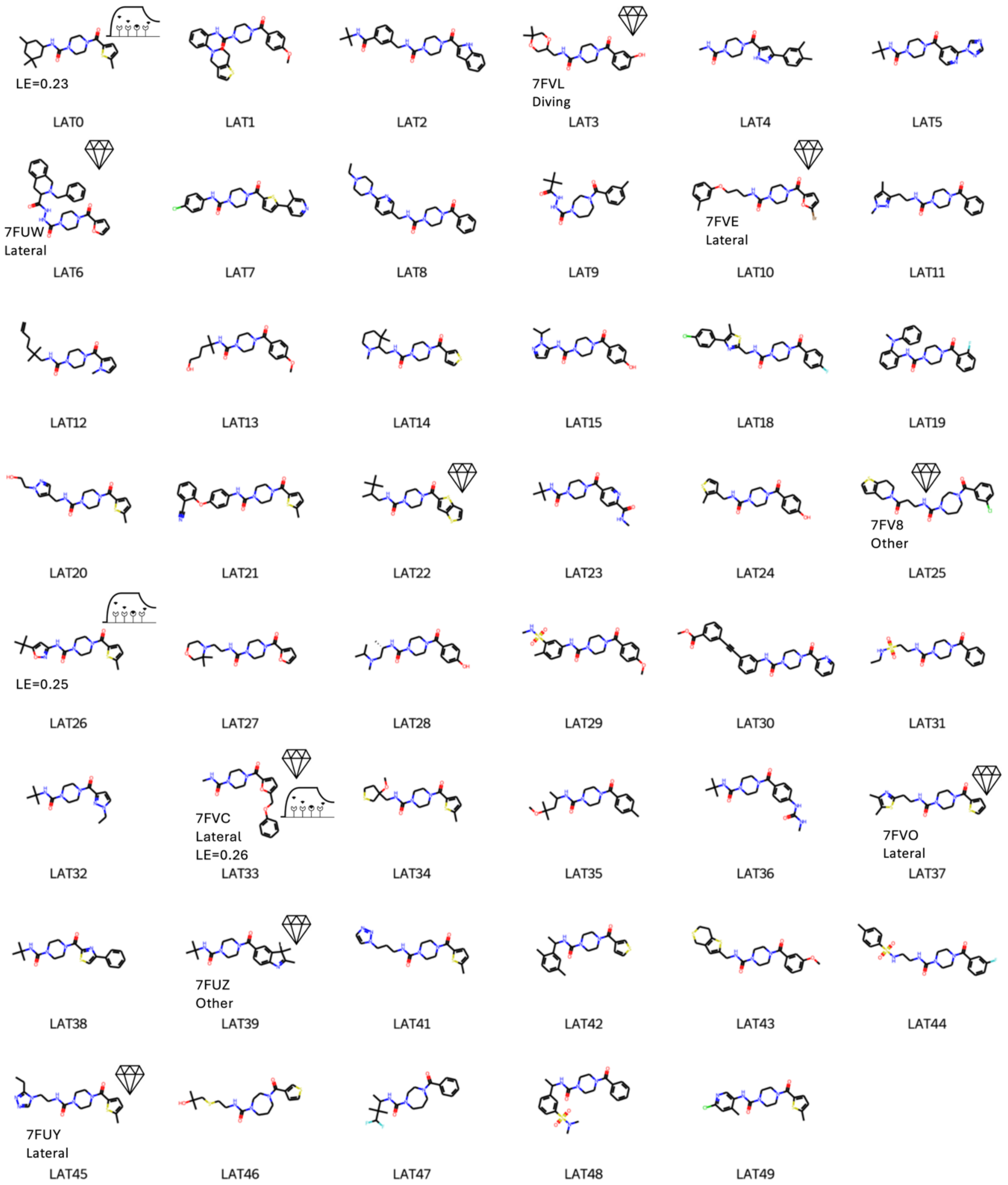

Supplementary figure 13: Virtual screening compounds for the lateral pose (part of the Prospective-93 set) purchased and screened by GCI and X-ray crystallography. The figure presents the chemical structures of 47 compounds selected from the virtual screening workflow targeting the lateral binding pose (making up a portion of the Prospective-93 dataset). These compounds were purchased in pure form and tested by X-ray crystallography against PHIP(2) to validate predictions made by the xSAR model. Each compound is labeled with its unique identifier (LATX) and, where applicable, the experimentally determined crystallographic binding mode ("Lateral," "Diving," or "Other"). Diamonds indicate crystallographically confirmed binders. Additionally, ligand efficiency (LE) values are provided for selected compounds to illustrate the efficiency of heavy atom contributions to binding affinity. The sensorgram icon denotes compounds classified as kinetic binders, as defined by the hit-calling criteria in the GCI assay.

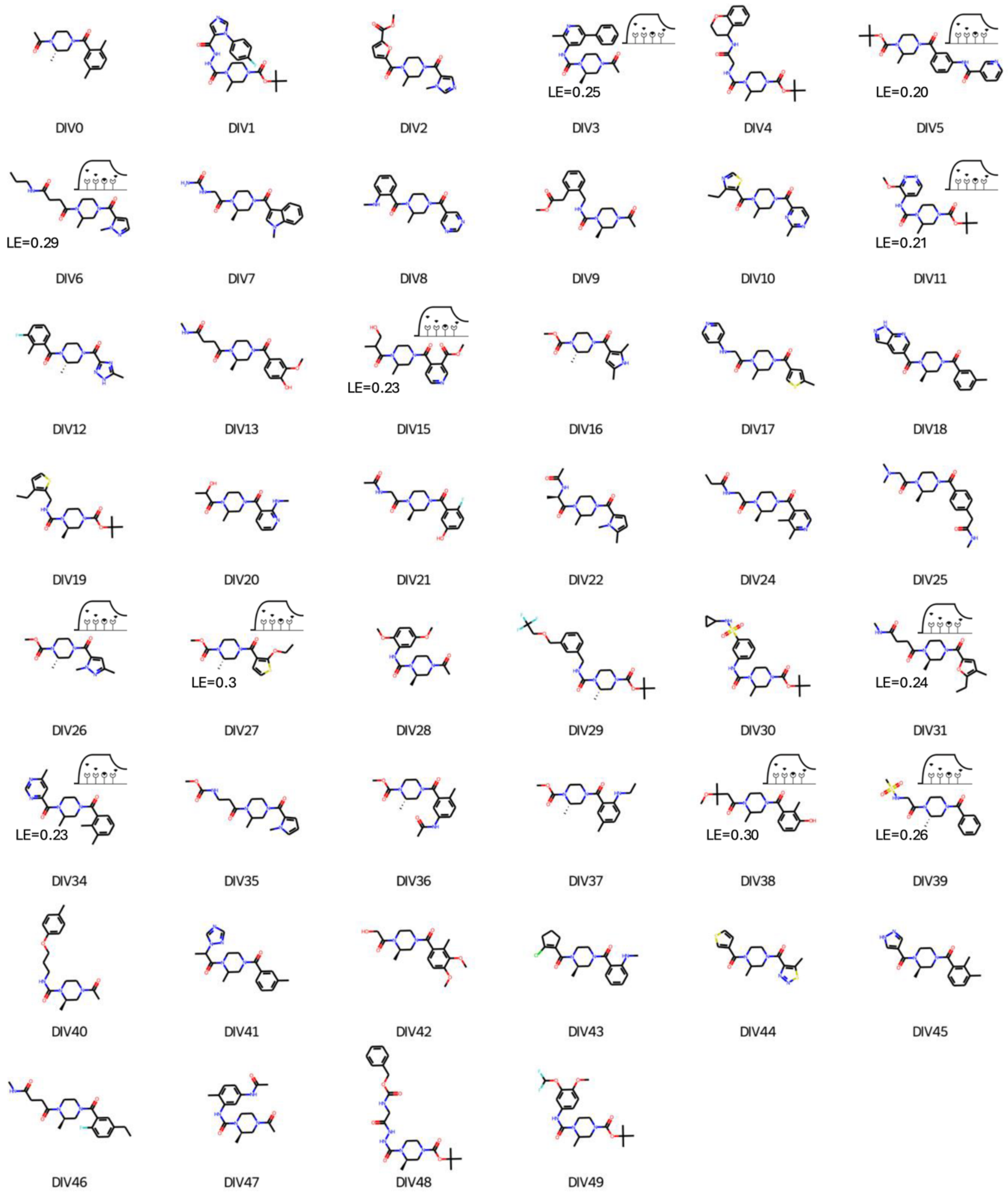

Supplementary figure 14: Virtual screening compounds for the diving pose (part of the Prospective-93 set) purchased and screened by GCI and X-ray crystallography. The figure presents the chemical structures of 46 compounds selected from the virtual screening workflow targeting the lateral binding pose (making up a portion of the Prospective-93 dataset). These compounds were purchased in pure form and tested by X-ray crystallography against PHIP(2) to validate predictions made by the xSAR model. Each compound is labeled with its unique identifier (DIVX) and, where applicable, the experimentally determined crystallographic binding mode ("Lateral," "Diving," or "Other"). Diamonds indicate crystallographically confirmed binders. Additionally, ligand efficiency (LE) values are provided for selected compounds to illustrate the efficiency of heavy atom contributions to binding affinity. The sensorgram icon denotes compounds classified as kinetic binders, as defined by the hit-calling criteria in the GCI assay.

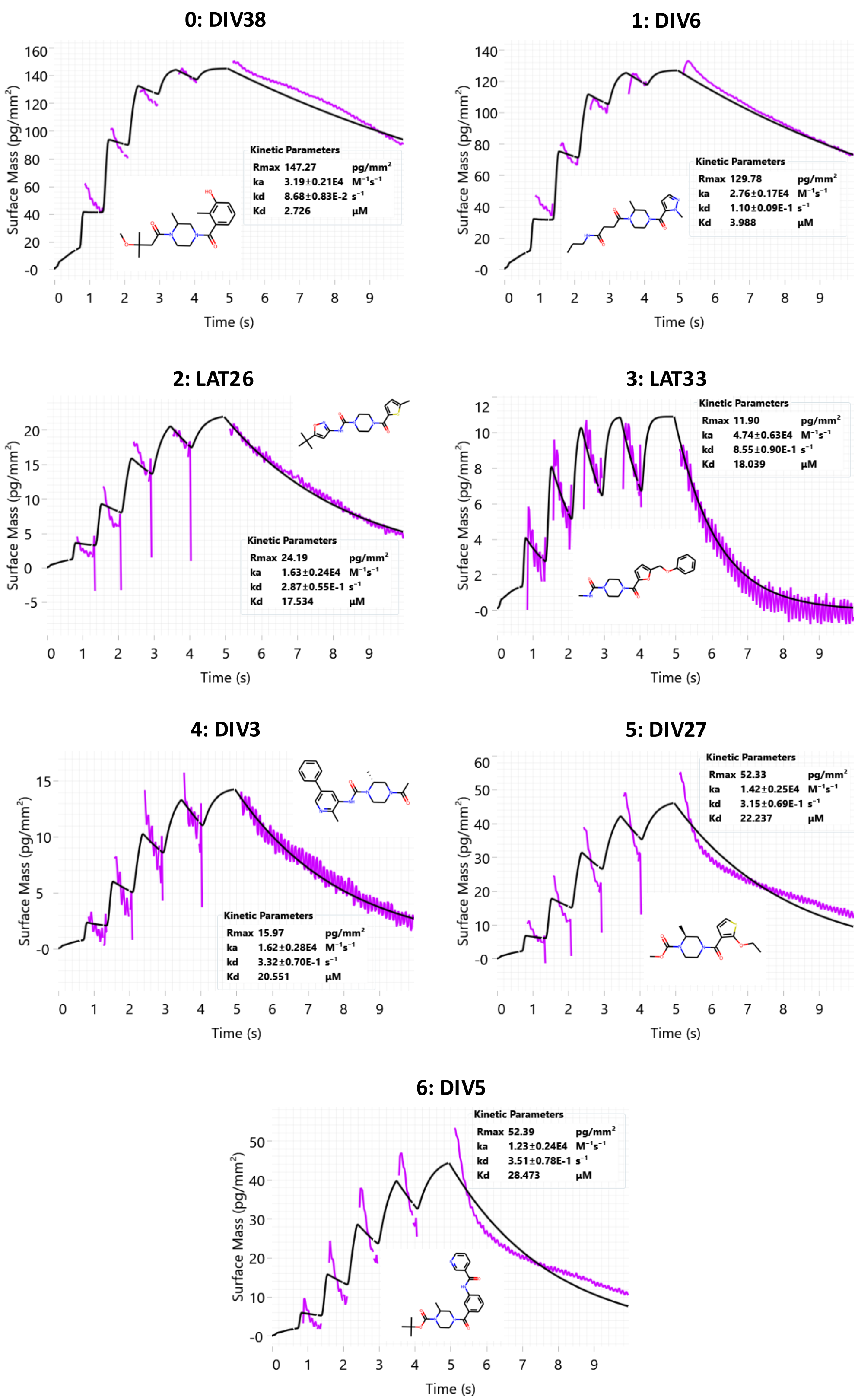

Supplementary figure 15.1: Kinetic binding curves for GCI hits from the virtual screen (Prospective-93 set). This table presents the binding curves obtained from GCI assays, with the black line representing the raw data and the fitted model shown in purple. The kinetic parameters, including association rate (k_a), dissociation rate (k_d), and equilibrium dissociation constant (Kd), were deduced from the fitted model, and are displayed in a separate box. The table also includes 2D molecule structures to illustrate the chemical structures of the molecules assayed.

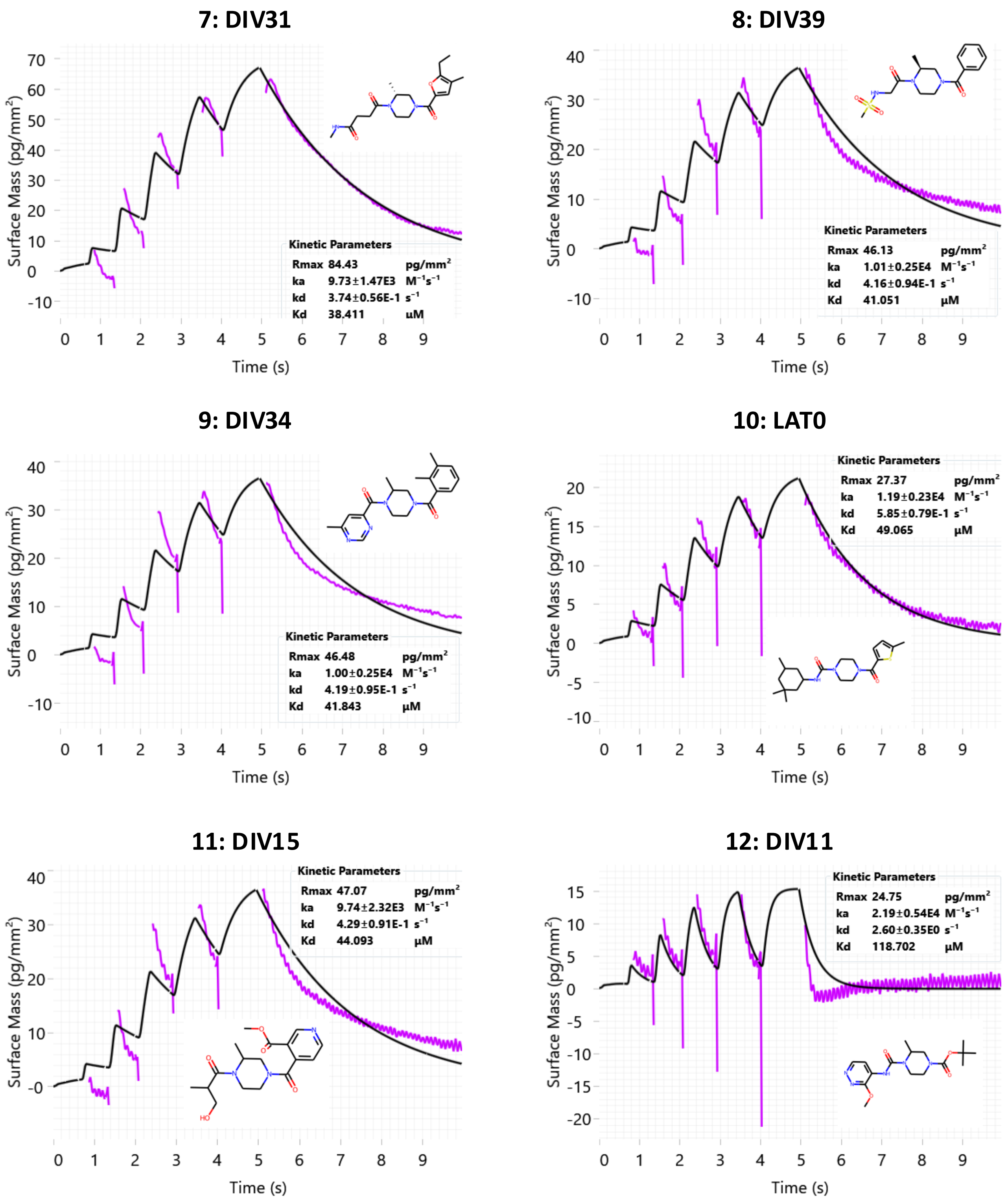

Supplementary figure 15.2: Kinetic binding curves for GCI hits from the virtual screen (Prospective-93 set). This table presents the binding curves obtained from GCI assays, with the black line representing the raw data and the fitted model shown in purple. The kinetic parameters, including association rate (k_a), dissociation rate (k_d), and equilibrium dissociation constant (Kd), were deduced from the fitted model, and are displayed in a separate box. The table also includes 2D molecule structures to illustrate the chemical structures of the molecules assayed.

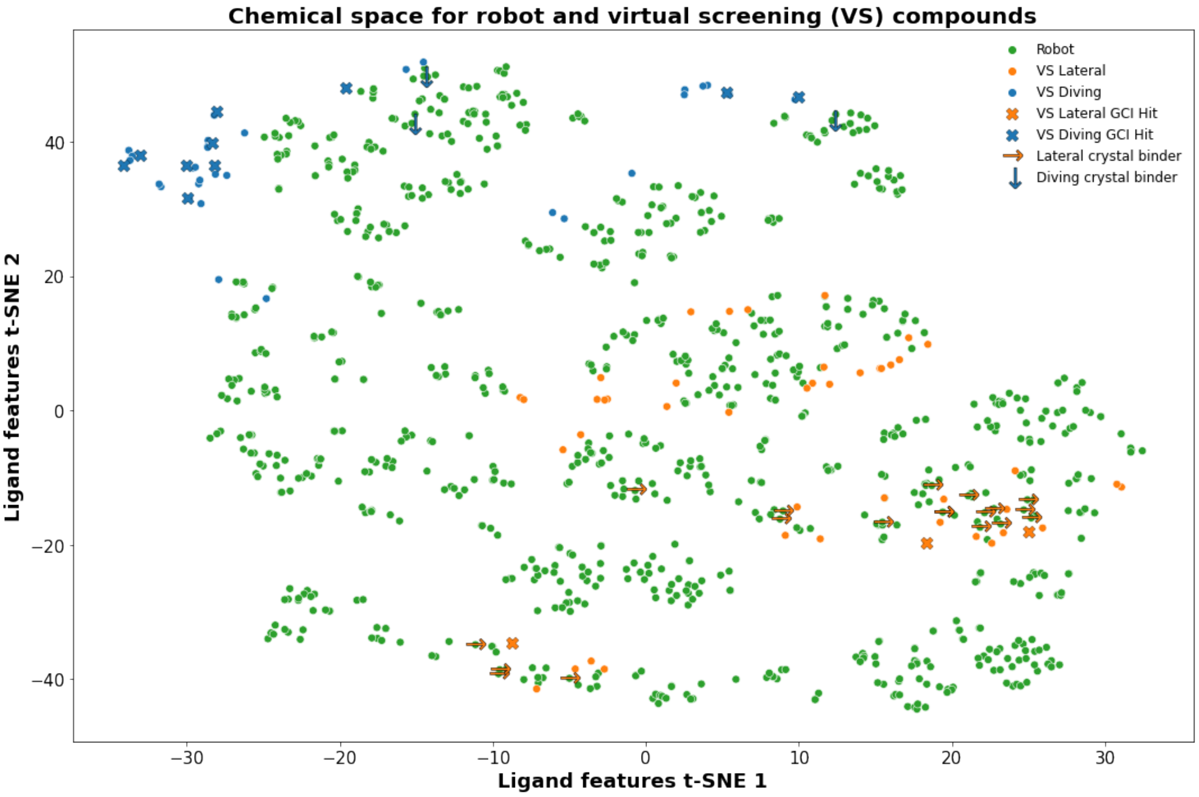

Supplementary figure 16: Prospective compounds and GCI hits mapped onto the robot-defined chemical space. The 2D chemical space was recomputed with t-SNE using Morgan fingerprints and now includes both the OriginalRefined-957 ("Robot") set and the Prospective-93 compounds; consequently, the layout is not identical to previous t-SNE plots because the dataset changed and t-SNE is stochastic. Green circles: robot compounds. Orange dots: prospective compounds selected with the lateral xSAR scores. Blue dots: prospective compounds selected with the diving xSAR scores. Orange and blue crosses: GCI hits from the lateral- and diving-scored sets, respectively. Rightward and downward arrows: crystallographic binders resolved in the lateral and diving pose, respectively. The arrow only show the initial CRM binders and not the false negatives as only those were used to build the xSAR scoring functions used in the virtual screening.

Supplementary future steps

Formalism for bit weighting

To downweight bits that occur frequently (e.g. aliphatic carbons)[1] within a compound (x_k), a weighting scheme ($\omega_m^{occ}(x_k)$), inspired from pseudocounts of intra-compound bit occurrences can be introduced as a sigmoid function:

$$\omega_m^{occ}(x_k) = \frac{1}{1 + \#(b_m \in x_k)} \qquad \text{Eq. S1}$$

Where $\#(b_m \in x_k)$ is the occurrence of bit b_m in compound x_k. This downweights bits that appear multiple times, reducing their contribution to conservation scores.

This weight is incorporated into the Binding Bit Conservation Score**:**

$$S_m^{\mathcal{B},\omega} = \frac{\sum_{\mathrm{x_i} \in \mathcal{B}} \omega_m^{occ}(\mathrm{x}_i)\mathrm{b_m}(\mathrm{x}_i)}{\sum_{\mathrm{x_i} \in \mathcal{B}} \omega_m^{occ}(\mathrm{x}_i)} \qquad \text{Eq. S2}$$

Where $\omega_m^{occ}(\mathrm{x}_i)$ represent the pseudocount-based bit weight. This approach may however amplify noise (i.e infrequent and coincidental bits that have no significant or negative impact on binding).

Formalism for additional bit conservation scores

Beyond the standard Binding Bit Conservation Score ($S_m^{\mathcal{B}}$), two additional scores can be introduced.

The Non-Binding Bit Conservation Score ($S_m^{\mathcal{NB}}$) measures bit conservation within non-binders:

$$S_m^{\mathcal{NB}} = \frac{\sum_{\mathrm{x_i} \in \mathcal{NB}} \mathrm{b_m}(\mathrm{x}_i)}{|\mathcal{NB}|} \qquad \text{Eq. S3}$$

As in Eq. S1 and Eq. S2, the Non-Binding Bit Conservation Score can be downweighted with intra-compound bit occurrences:

$$S_m^{\mathcal{NB},\omega} = \frac{\sum_{\mathrm{x_i} \in \mathcal{NB}} \omega_m^{occ}(\mathrm{x}_i)\mathrm{b_m}(\mathrm{x}_i)}{\sum_{\mathrm{x_i} \in \mathcal{NB}} \omega_m^{occ}(\mathrm{x}_i)} \qquad \text{Eq. S4}$$

The Overall Bit Conservation Score ($S_m^{\mathcal{D}}$) measures bit conservation across all compounds.

$$S_m^{\mathcal{D}} = \frac{\sum_{\mathrm{x_i} \in \mathcal{D}} \mathrm{b_m}(\mathrm{x}_i)}{|\mathcal{D}|} \qquad \text{Eq. S5}$$

As in Eq. S1 and Eq. S2, the Overall Bit Conservation Score can be downweighted with intra-compound bit occurrences:

$$S_m^{\mathcal{D},\omega} = \frac{\sum_{\mathrm{x_i} \in \mathcal{D}} \omega_m^{occ}(\mathrm{x}_i)\mathrm{b_m}(\mathrm{x}_i)}{\sum_{\mathrm{x_i} \in \mathcal{D}} \omega_m^{occ}(\mathrm{x}_i)} \qquad \text{Eq. S6}$$

Formalism for bit classification by thresholding

The use of the novel bit conservation scores $S_m^{\mathcal{NB}}$ and $S_m^{\mathcal{D}}$, weighted or not, to the previously defined Binding Bit Conservation Score ($S_m^{\mathcal{B}}$, Eq. 1) enable a new bit classification scheme enable by the usage the conservation thresholds $\theta_{\mathcal{CBB}}$ and $\theta_{\mathcal{CNB}}$. The threshold $\theta_{\mathcal{CBB}}$ and $\theta_{\mathcal{CNB}}$ correspond to the bit recovery rate required to classify a bit into $\mathcal{CBB}$ or $\mathcal{CNB}$ defining the minimum fraction of compounds in binders ($\mathcal{B}$) and non-binders ($\mathcal{NB}$) in which a bit must be active to be assigned to a specific category, respectively.

Each bit, $\mathrm{b_m}$, is assigned to one of four mutually exclusive categories based on its conservation scores:

The Conserved Binding Bits ($\mathcal{CBB}$) can now be defined as:

$$\mathrm{b_m} \in \mathcal{CBB} \iff S_m^{\mathcal{B}} \geq \theta_{\mathcal{CBB}}$$

meaning that bit, $\mathrm{b_m}$, is highly conserved in binders, meaning it appears in at least a fraction $\theta_{\mathcal{CBB}}$of the compounds classified as binders.

The Conserved Non-binding Bits ($\mathcal{CNB}$) can now be defined as:

$$\mathrm{b_m} \in \mathcal{CNB} \iff S_m^{\mathcal{B}} < \theta_{\mathcal{CBB}} \; and \; S_m^{\mathcal{NB}} \geq \theta_{\mathcal{CNB}}$$

meaning that bit, $\mathrm{b_m}$, is not conserved in binders (below $\theta_{\mathcal{CBB}}$), but it is conserved in non-binders, meaning it appears in at least fraction $\theta_{\mathcal{CNB}}$ of non-binders.

The Unconserved Bits ($\mathcal{UCB}$) can now be defined as:

$$\mathrm{b_m} \in \mathcal{UCB} \iff S_m^{\mathcal{B}} < \theta_{\mathcal{CBB}} \; and \; S_m^{\mathcal{NB}} < \theta_{\mathcal{CNB}} \; and \; S_m^{\mathcal{D}} > 0$$

meaning that bit, $\mathrm{b_m}$, is is not conserved in either binders or non-binders but still appears at least once in the dataset.

The Unsampled Bits ($\mathcal{USB}$) can now be defined as:

$$\mathrm{b_m} \in \mathcal{USB} \iff S_m^{\mathcal{D}} = 0$$

meaning that bit, $\mathrm{b_m}$, is inactive across all compounds.

Formalism for score weighting

Similarly to Eq. S1 and Eq. S2, a pseudocount-based weight, using for example the Overall Bit Conservation Score ($S_m^{\mathcal{D}}$), can be implement as a sigmoid function to

downweigh frequently occurring and therefore less specific bit appearing across compounds.

$$\omega_m^{occ} = \frac{1}{1 + S_m^{\mathcal{D}}} \quad \text{Eq. S7}$$

Again, this approach may amplify noise (i.e infrequent and coincidental bits that have no significant or negative impact on binding).

It may also be possible to include structural, dynamic and energetic context in the scoring scheme by assigning a weight based on the relative binding free energy ($\Delta\Delta G$) associated with removing the substructure corresponding to bit $\mathrm{b_m}$ within cocrystalised or docked compound $\mathrm{x_k}$, assuming the bit $\mathrm{b_m}$ exist uniquely within compound $\mathrm{x_k}$.

$$\omega_m^{\Delta G} = \frac{1}{1 + \mathrm{e}^{\Delta\Delta G_m}} \quad \text{Eq. S8}$$

Where $\Delta\Delta G_m$ represents the change in binding free energy upon removal of the substructure corresponding to bit $\mathrm{b_m}$ and $\omega_m^{\Delta G}$ is the free energy-based weight for bit $\mathrm{b_m}$. If $\Delta\Delta G_m < 0$ (removal improves binding), $\mathrm{b_m}$ is upweighted. If $\Delta\Delta G_m > 0$ (removal weakens binding) $\mathrm{b_m}$ is downweighted. If $\Delta\Delta G_m = 0$ (removal does not affect binding $\mathrm{b_m}$ remains neutral).

Then, those weights could be included in the binding scores similarly to Eq. S2. Hence the Positive Binding Score (PBS) for compound $\mathrm{x_k}$ would become:

$$\mathrm{PBS}(\mathrm{x_k}) = \frac{\sum_{\mathrm{b_m} \in \mathcal{CBB}} \omega_m \mathrm{b_m}(\mathrm{x}_k)}{\sum_{\mathrm{b_m} \in \mathcal{CBB}} \omega_m(\mathrm{x}_k)} \quad \text{Eq. S9}$$

And the Negative Binding Score NBS for compound $\mathrm{x_k}$ would become:

$$\mathrm{NBS}(\mathrm{x_k}) = 1 - \frac{\sum_{\mathrm{b_m} \in \mathcal{CNB}} \omega_m \mathrm{b_m}(\mathrm{x}_k)}{\sum_{\mathrm{b_m} \in \mathcal{CNB}} \omega_m(\mathrm{x}_k)} \quad \text{Eq. S10}$$

With ω_m being any weighting scheme such as the ω_m^{occ} or $\omega_m^{\Delta G}$defined above in Eq. S7 and Eq. S8.

Formalism for ensemble scores

Both PBS and NBS, weighted or not, can be combined into one scoring scheme $\mathrm{E(x_k)}$ to account for both binding and non-binding features simultaneously.

$$\mathrm{E(x_k)} = \lambda \cdot \mathrm{PBS(x_k)} + (1 - \lambda) \cdot \mathrm{NBS(x_k)} \qquad \text{Eq. S11}$$

Where $\lambda \in [0, 1]$ is a scaling parameter that determines the relative contribution of PBS and NBS. This would also imply that a single threshold θ_E would be needed to categorise likely and unlikely binders where:

$$\mathrm{E(x_k)} \geq \theta_E \;\Rightarrow predicted\ binder$$

$$\mathrm{E(x_k)} < \theta_E \;\Rightarrow predicted\ nonbinder$$

Supplementary methods

Benchmarking of ligand-based classification methods

To test the performance of the PBS and NBS metrics on discriminating binders from non-binders, they were benchmarked against other commonly used ligand-based classification approaches. The task was to classify a compound as either a binder or non-binder from its features. The training set was the OriginalRefined-957 set excluding compounds from the Retrospective-97 set, totaling 860 compounds. The test set was the Retrospective-97 set with the binding labels measured from the rescreening experiment. The conserved binding and non-binding bits to formulate the PBS and NBS metrics were extracted from the training set only.

The benchmark included a random forest classifier with hyperparameters optimised against the test set and two Tanimoto similarity scores on the Morgan fingerprints.

Tanimoto coefficient classifiers

Two Tanimoto chemical similarity distance-based scores were also included in the benchmark. To quantify the structural similarity between test compounds and known binders, we computed the mean and maximum Tanimoto coefficients (TC) relative to the reference set of known binders. Morgan fingerprints were generated with RDKit[2] (radius = 6, 2048-bit length) with feature-based encoding enabled. For each test molecule, the TC was computed against all known binders in the training set, and either the mean or maximum value was compared.

Random forest hyperparameters training, optimisation and classification

The random forest model was trained using the pose specific OriginalRefined-957 dataset excluding the Retrospective-97 datapoints, which served as the training set. The Retrospective-97 subset was held out entirely, during training, and used as the test set to assess model performance.

The hyperparameters for the random forest model were optimised across all three data subsets of the test set employing the Optuna library and its Tree-structured Parzen Estimator algorithm[3]. The optimisation process comprised 1,000 initial trials followed by a total of 2,500 trials. Four hyperparameters were optimised: maximum tree depth, maximum number of features used at each split, class weight, and number of trees in the forest. The maximum depth was evaluated within a range of 1 to 40 units with a step size of 1, while the maximum features values were assessed between "None" and various options, including "sqrt", "log2", and float values from 0.6 to 1.0 with a step size of 0.1. The class weight was examined among four distinct options: None, "balanced", {0:1, 1:10}, and {0:1, 1:100}. Class 0 symbolises negatives or non-binders, and class 1 represents positives or binders. The number of trees in the forest was explored from 10 to 1,000 with a step size of 50. The objective criterion aimed to maximise the average precision-recall area under the curve across 20 repeats calculated with scikit-learn.

Random forest classifications were finally applied to the same test set using scikit-learn, employing the best-identified hyperparameters. The average precision-recall area under the curve across 200 repeats was recorded to evaluate performance. To compare model performances between raw and corrected datasets, leave-one-out cross-validations were executed in triplicate for all datasets and average precision-recall AUC recorded and utilised to assess performance.

Statistical analysis

The Mann-Whitney U test[4], implemented using the SciPy library, was chosen for testing how significantly enriched the binding scores the identification of binders over non-binder during the rescreening experiment. Due to the limited sample size, independent nature of the data points (different ligands were screened) and the non-normal binding score distributions, a non-parametric statistical test was employed. The

test was conducted with a two-sided alternative hypothesis to assess the significance of difference of binding scores between the two groups.

The predictions generated by the different classifiers (PBS, NBS, random forest, mean TC, max TC) were ranked thus allowing for calculation of the precision-recall AUC (PR-AUC) (Fig. S11). The precision-recall area under the curve accounts for class imbalance and is therefore superior to receiver operating characteristic curve[5]. The precision and recall correspond to the positive predictive value (PPV) and true positive rate, respectively.

The precision is calculated as:

$$PPV = \frac{TP}{TP + FP} \quad \text{Eq. S12}$$

The recall is calculated as:

$$TPR = \frac{TP}{TP + FN} \quad \text{Eq. S13}$$

Where TP, FP and FN correspond to the number of true positives (correctly classified positive cases), false positives (incorrectly classified positive cases), and false negatives (incorrectly classified negative cases), respectively. The precision-recall area under the curve value can be estimated by calculating the precision and recall values across possible threshold values. The precision-recall area under the curve is calculated as:

$$PR\ AUC = \int_0^1 PPV(TPR)dTPR \quad \text{Eq. S14}$$

This integral was estimated using the trapezoidal rule implemented in scikit-learn.

Supplementary references

(1) Arif, S. M.; Holliday, J. D.; Willett, P. Inverse Frequency Weighting of Fragments for Similarity-Based Virtual Screening. *J. Chem. Inf. Model.* **2010**, *50* (8), 1340–1349. https://doi.org/10.1021/ci1001235.

(2) Landrum, G.; Tosco, P.; Kelley, B.; Ric; Cosgrove, D.; Sriniker; Gedeck; Vianello, R.; NadineSchneider; Kawashima, E.; N, D.; Jones, G.; Dalke, A.; Cole, B.; Swain, M.; Turk, S.; AlexanderSavelyev; Vaucher, A.; Wójcikowski, M.; Ichiru Take; Probst, D.; Ujihara, K.; Scalfani, V. F.; Godin, G.; Lehtivarjo, J.; Walker, R.; Pahl, A.; Francois Berenger; Jasondbiggs; Strets123. Rdkit/Rdkit: 2023_03_3 (Q1 2023) Release, 2023. https://doi.org/10.5281/ZENODO.8254217.

(3) Akiba, T.; Sano, S.; Yanase, T.; Ohta, T.; Koyama, M. Optuna: A Next-Generation Hyperparameter Optimization Framework. In *Proceedings of the 25th ACM SIGKDD International Conference on Knowledge Discovery & Data Mining*; KDD '19; Association for Computing Machinery: New York, NY, USA, 2019; pp 2623–2631. https://doi.org/10.1145/3292500.3330701.

(4) McKnight, P. E.; Najab, J. Mann-Whitney U Test. In *The Corsini Encyclopedia of Psychology*; John Wiley & Sons, Ltd, 2010; pp 1–1. https://doi.org/10.1002/9780470479216.corpsy0524.

(5) Saito, T.; Rehmsmeier, M. The Precision-Recall Plot Is More Informative than the ROC Plot When Evaluating Binary Classifiers on Imbalanced Datasets. *PLOS ONE* **2015**, *10* (3), e0118432. https://doi.org/10.1371/journal.pone.0118432.
